# Supplementary material for: Quantifying massively parallel microbial growth with spatially mediated interactions
Source: PLoS Comput Biol. 2024 Jul 22;20(7):e1011585. doi: 10.1371/journal.pcbi.1011585 (PMC11293690; doi:10.1371/journal.pcbi.1011585)
Supplement: S1 Table — All error rates displayed for all 4 environments are obtained by using the SSEs between the predictions of the respective models for the values of ρ^i(t) and the ρi(t) values calculated from the experimental data. Similarly, the main models are compared as against the null model using AIC (smaller value is better). The scores indicate that the diffusion model is the best model for all environments except Gal + NaCl where AIC would choose the density-dependent model. However, as the density-dependent model has the spatial component modelled with a “black box” of ϵk(t) we prefer the fully mechanistic diffusion model also for the salt-containing galactose environment. (PDF) [file pcbi.1011585.s001.pdf]

S1 Table. Master table.

Model	Variables	Associated number of parameters	Dimensionality
ML without location	$N_i(t)$	(implementation-dependent)	?
ML with location	row, col, $N_i(t)$	(implementation-dependent)	?
null model	$\rho_i(t)$	218	218
population-specific	r_0, m, c_i, ϵ	1, 1, 1536, 218	1756
location-and-population-specific	r_0, m, c_i, ϵ_k	1, 1, 1536, 16 * 218	5026
density-dependent	r_0, m, c_i, ϵ_k, ν	1, 1, 1536, 16 * 218, 1	5027
diffusion with $\nu_2 = 0$	r_0, m, c_i, ν, D, K, κ	1, 1, 1536, 1, 1, 1, 1	1542
diffusion model	r_0, m, c_i, ν_1, ν_2, D, K, κ	1, 1, 1536, 1, 1, 1, 1, 1	1543

Model	Fitting error (sum of squared errors)				Comparison with null model (AIC)			
	Glc	Glc + NaCl	Gal	Gal + NaCl	Glc	Glc + NaCl	Gal	Gal + NaCl
ML without location	0.70	0.93	1.00	1.17				
ML with location	0.08	0.21	0.12	0.20				
null model	4.56	3.83	4.50	5.57				
population-specific	3.82	3.40	3.37	2.66	-4.91 * 10^4	-3.51 * 10^4	-9.05 * 10^4	-2.33 * 10^5
location-and-population	2.12	2.87	1.93	1.87	-2.34 * 10^5	-8.25 * 10^4	-2.61 * 10^5	-3.39 * 10^5
density-dependent	1.42	1.51	1.10	0.78	-3.65 * 10^5	-2.99 * 10^5	-4.36 * 10^5	-6.04 * 10^5
diffusion with $\nu_2 = 0$	28.99	8.23	13.03	3.92				
diffusion model	1.02	0.88	0.72	1.10	-4.82 * 10^5	-4.66 * 10^5	-5.85 * 10^5	-5.21 * 10^5

Master table. All error rates displayed for all 4 environments are obtained by using the SSEs between the predictions of the respective models for the values of $\hat{\rho}_i(t)$ and the $\rho_i(t)$ values calculated from the experimental data. Similarly, the main models are compared as against the null model using AIC (smaller value is better). The scores indicate that the diffusion model is the best model for all environments except Gal + NaCl where AIC would choose the density-dependent model. However, as the density-dependent model has the spatial component modelled with a "black box" of $\epsilon_k(t)$ we prefer the fully mechanistic diffusion model also for the salt-containing galactose environment.
