# Supplementary material for: Quantifying massively parallel microbial growth with spatially mediated interactions
Source: PLoS Comput Biol. 2024 Jul 22;20(7):e1011585. doi: 10.1371/journal.pcbi.1011585 (PMC11293690; doi:10.1371/journal.pcbi.1011585)
Supplement: S2 Fig — Each subfigure represents, for each plate, the sum of squared errors (SSE) between the relative growth rates ρi(t), calculated from the data obtained by simulation, and computed rates ρ^i(t), as predicted by our models, namely: 1) the null model, which consists of simply averaging for every time point the growth rates across a plate, 2) the αϵ model, which represents the αi(t)ϵ(t) model where the latter component is a mechanism-free parameter, 3) the αϵk model, which represents the αi(t)ϵk(t) model where the mechanism-free approach relaxes the global constraint of the parameter, 4) the αϵks model, which adds a nutrient consumption term, 5) the diffusion model, which further removes the mechanism-free parameter and represents its effect through a diffusion process, and 6) the random forest model, which consists of a RandomForestRegressor from scikit-learn trained on 75% of the data and then predicting 25% of the growth rates by using the location on the plate and the current population size as input features. A—D The spatial representation of the fitting errors, where the SSE are computed for each population individually for all the time points. Here, rhe predictions made by the random forest model are obtained for both the training and testing data. E The temporal representation of the fitting errors, where the SSE are computed for all the populations of a plate at every time point. Here, the predictions made by the random forest model are obtained for both the training and testing data. (PDF) [file pcbi.1011585.s003.pdf]

S2 Fig. Results for the synthetic dataset.

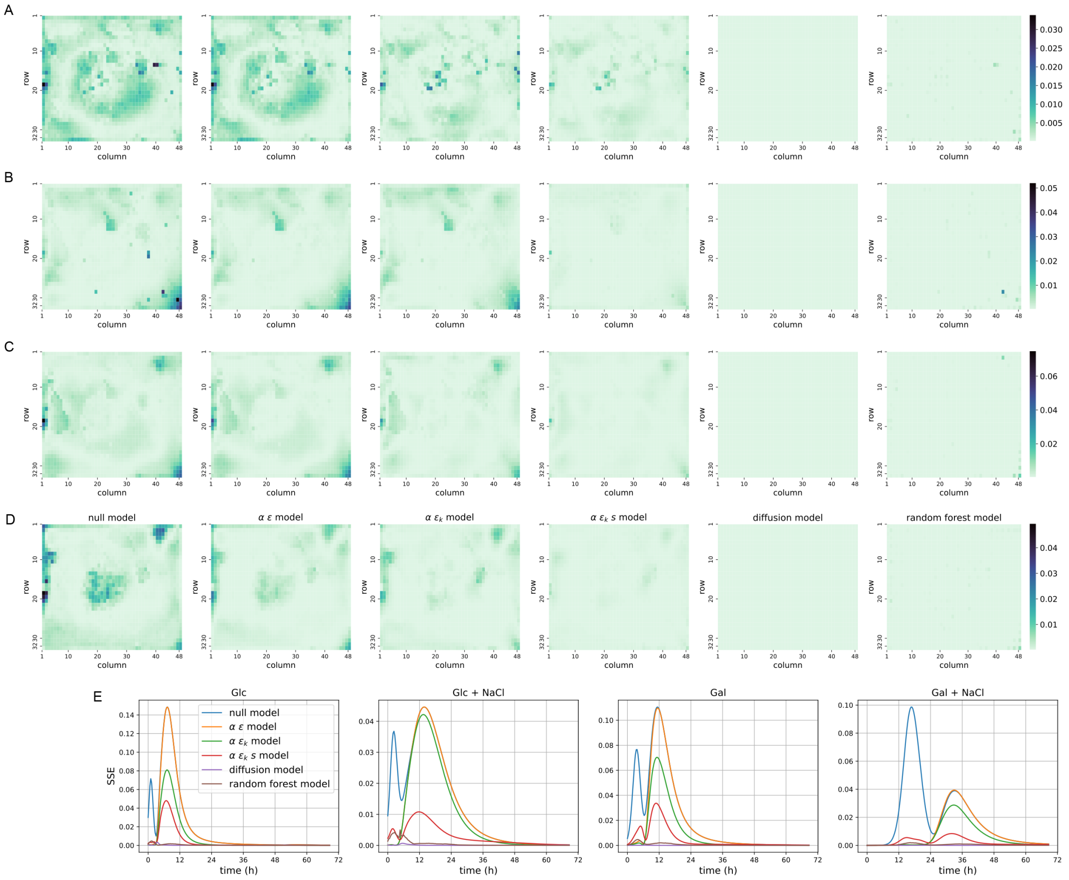

Each subfigure represents, for each plate, the sum of squared errors (SSE) between the relative growth rates $\rho_i(t)$, calculated from the data obtained by simulation, and computed rates $\hat{\rho}_i(t)$, as predicted by our models, namely: 1) the null model, which consists of simply averaging for every time point the growth rates across a plate, 2) the $\alpha\epsilon$ model, which represents the $\alpha_i(t)\epsilon(t)$ model where the latter component is a mechanism-free parameter, 3) the $\alpha\epsilon_k$ model, which represents the $\alpha_i(t)\epsilon_k(t)$ model where the mechanism-free approach relaxes the global constraint of the parameter, 4) the $\alpha\epsilon_k s$ model, which adds a nutrient consumption term, 5) the diffusion model, which further removes the mechanism-free parameter and represents its effect through a diffusion process, and 6) the random forest model, which consists of a RandomForestRegressor from scikit-learn trained on 75 % of the data and then predicting 25 % of the growth rates by using the location on the plate and the current population size as input features.

A - D The spatial representation of the fitting errors, where the SSE are computed for each population individually for all the time points. Here, rhe predictions made by the random forest model are obtained for both the training and testing data.

E The temporal representation of the fitting errors, where the SSE are computed for all the populations of a plate at every time point. Here, the predictions made by the random forest model are obtained for both the training and testing data.
