# Supplementary material for: Quantifying massively parallel microbial growth with spatially mediated interactions
Source: PLoS Comput Biol. 2024 Jul 22;20(7):e1011585. doi: 10.1371/journal.pcbi.1011585 (PMC11293690; doi:10.1371/journal.pcbi.1011585)
Supplement: S3 Fig — The diffusion model introduced in this article uses 7 global parameters—r0 and m are involved in the αi(t) term, D, ν1 and ν2 are involved in the calculation of ∂s/∂t, and K and κ are involved in the f(s) function—which are inferred by fitting the model on two different data sets: first the Scan-o-matic data, labelled here as experimental, and then the same synthetic data set as in S2 Fig which was simulated with the real data inferred parameters. The inferred synthetic parameters are within a reasonable range from the inferred experimental parameters thus demonstrating veracity and self-consistency of the inference approach. This is true in particular for the parameters r0, m, D and ν1, while the other three, ν2, K and κ show a degree of interplay. (PDF) [file pcbi.1011585.s004.pdf]

## S3 Fig. Obtained diffusion model parameters.

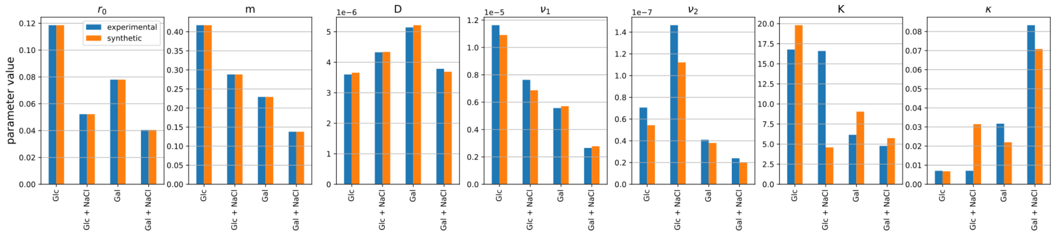


**Comparison of fitted diffusion model parameters between the Scan-o-matic data and the synthetic data.** The diffusion model introduced in this article uses 7 global parameters – $r_0$ and $m$ are involved in the $\alpha_i(t)$ term, $D$, $\nu_1$ and $\nu_2$ are involved in the calculation of $\partial s/\partial t$, and $K$ and $\kappa$ are involved in the $f(s)$ function – which are inferred by fitting the model on two different data sets : first the Scan-o-matic data, labelled here as experimental, and then the same synthetic data set as in S2 which was simulated with the real data inferred parameters.

The inferred synthetic parameters are within a reasonable range from the inferred experimental parameters thus demonstrating veracity and self-consistency of the inference approach. This is true in particular for the parameters $r_0$, $m$, $D$ and $\nu_1$, while the other three, $\nu_2$, $K$ and $\kappa$ show a degree of interplay.
