# Supplementary material for: Quantifying massively parallel microbial growth with spatially mediated interactions
Source: PLoS Comput Biol. 2024 Jul 22;20(7):e1011585. doi: 10.1371/journal.pcbi.1011585 (PMC11293690; doi:10.1371/journal.pcbi.1011585)
Supplement: S4 Fig — The adjustment function α(t) depends on three parameters r0, m and c. Throughout the article, we chose to make the former two parameters global across a plate, while the latter was made local to a population i. By generating synthetic population growth data according to our diffusion model parameters, but setting ci=c¯i (plate mean) for all populations of a plate, we can contrast the results from Fig 2A by using a similar ML-based approach on the generated synthetic data. A A synthetic data set where all 32x48 populations across a plate are simulated. While the general patterns between relative importances of population size Ni(t) and location obey to the same dynamics, there is a notable difference for the initial phase. The removal the physiological state related variability, by forcing a global α(t) adjustment function, removes the spatial feature being important early on. B A similar synthetic data set, but where only every fourth population is present—other locations are left unoccupied—by subdividing the 32x48 grid into 2x2 lattices, and keeping only the lower-right corner. The dynamics of the relative feature importances follow a similar pattern as for the full plate but as expected the spatial component, which reports diffusion, start to grow later. (PDF) [file pcbi.1011585.s005.pdf]

S4 Fig. Mean $\alpha(t)$ adjustment function parameters.

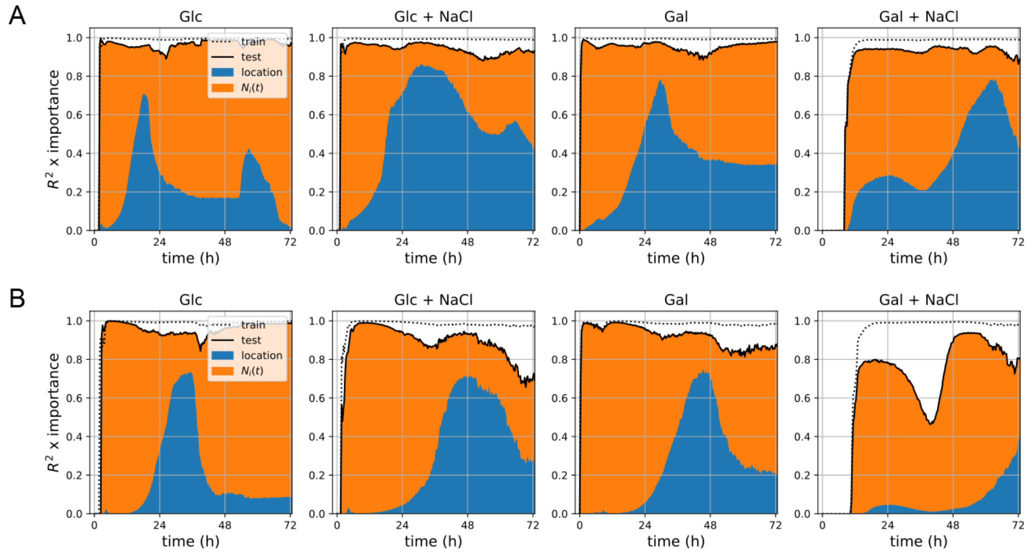

S4 Fig. Mean $\alpha(t)$ adjustment function parameters. The adjustment function $\alpha(t)$ depends on three parameters r_0, m and c. Throughout the article, we chose to make the former two parameters global across a plate, while the latter was made local to a population i. By generating synthetic population growth data according to our diffusion model parameters, but setting $c_i = \bar{c}_i$ (plate mean) for all populations of a plate, we can contrast the results from Fig 2A by using a similar ML-based approach on the generated synthetic data.

A A synthetic data set where all 32x48 populations across a plate are simulated. While the general patterns between relative importances of population size $N_i(t)$ and location obey to the same dynamics, there is a notable difference for the initial phase. The removal the physiological state related variability, by forcing a global $\alpha(t)$ adjustment function, removes the spatial feature being important early on.

B A similar synthetic data set, but where only every fourth population is present – other locations are left unoccupied – by subdividing the 32x48 grid into 2x2 lattices, and keeping only the lower-right corner. The dynamics of the relative feature importances follow a similar pattern as for the full plate but as expected the spatial component, which reports diffusion, start to grow later.
