# Supplementary material for: Quantifying massively parallel microbial growth with spatially mediated interactions
Source: PLoS Comput Biol. 2024 Jul 22;20(7):e1011585. doi: 10.1371/journal.pcbi.1011585 (PMC11293690; doi:10.1371/journal.pcbi.1011585)
Supplement: S5 Fig — Spatial patterns of population growth on semi-solid nutrient medium. A total of 1536 isogenic populations were pinned in a 32 x 48 grid onto each of 4 plates, containing semi-solid nutrient media with small variations, and cultivated for 72 h with measurements of population size taken every 20 min. The nutrient media of the plates contain respectively 2% glucose only, 2% glucose + 1 M NaCl as growth-limiting substrate, 2% galactose only, and 2% galactose + 1 M NaCl (data from [10]). A Plate averages of population size estimates Ni(t), absolute growth rates ΔNi(t) and relative, per capita, growth rates ρi(t), at each of the 218 time points. B Population size Ni(t) for all populations on each plate, coloured by layer of equivalent distances to the nearest grid border. Darker curves are closer to the border and exhibit greater growth. (PDF) [file pcbi.1011585.s006.pdf]

S5 Fig. Logarithmic representation of the growth data.

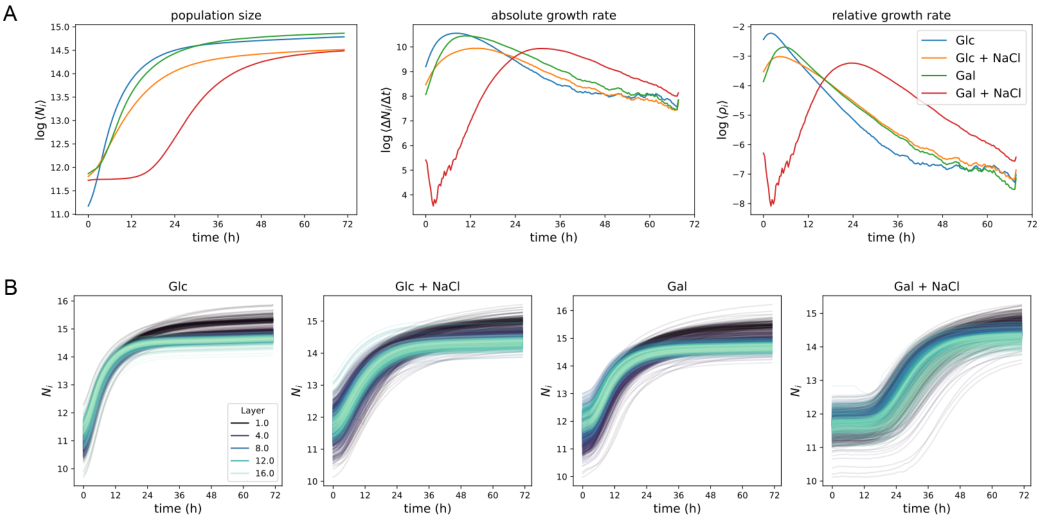

Spatial patterns of population growth on semi-solid nutrient medium. A total of 1536 isogenic populations were pinned in a 32 x 48 grid onto each of 4 plates, containing semi-solid nutrient media with small variations, and cultivated for 72 h with measurements of population size taken every 20 min. The nutrient media of the plates contain respectively 2 % glucose only, 2 % glucose + 1 M NaCl as growth-limiting substrate, 2 % galactose only, and 2 % galactose + 1 M NaCl.

A Plate averages of population size estimates $N_i(t)$, absolute growth rates $\Delta N_i(t)$ and relative, per capita, growth rates $\rho_i(t)$, at each of the 218 time points.

B Population size $N_i(t)$ for all populations on each plate, coloured by layer of equivalent distances to the nearest grid border. Darker curves are closer to the border and exhibit greater growth.
