# Supplementary material for: Quantifying massively parallel microbial growth with spatially mediated interactions
Source: PLoS Comput Biol. 2024 Jul 22;20(7):e1011585. doi: 10.1371/journal.pcbi.1011585 (PMC11293690; doi:10.1371/journal.pcbi.1011585)
Supplement: S6 Fig — The growth experiments have been run on two sources of nutrient, glucose and galactose. Additionally, both nutrients have been tested in conjunction with salt or without. A The nutrient diffusion D(s¯-s) term averaged per layer as in Fig 4A. The 16 layers are for both the salt-free and salt-containing environments represented as a curve getting darker the further the layer is from the grid border. B The respective contributions to nutrient consumption of population growth and population maintenance, as in Fig 4B. The colours are chosen to group curves according to their consumption term. That is, blue and green for population growth, and orange and red for population maintenance. (PDF) [file pcbi.1011585.s007.pdf]

S6 Fig. Comparisons between salt-free and salt-containing environments.

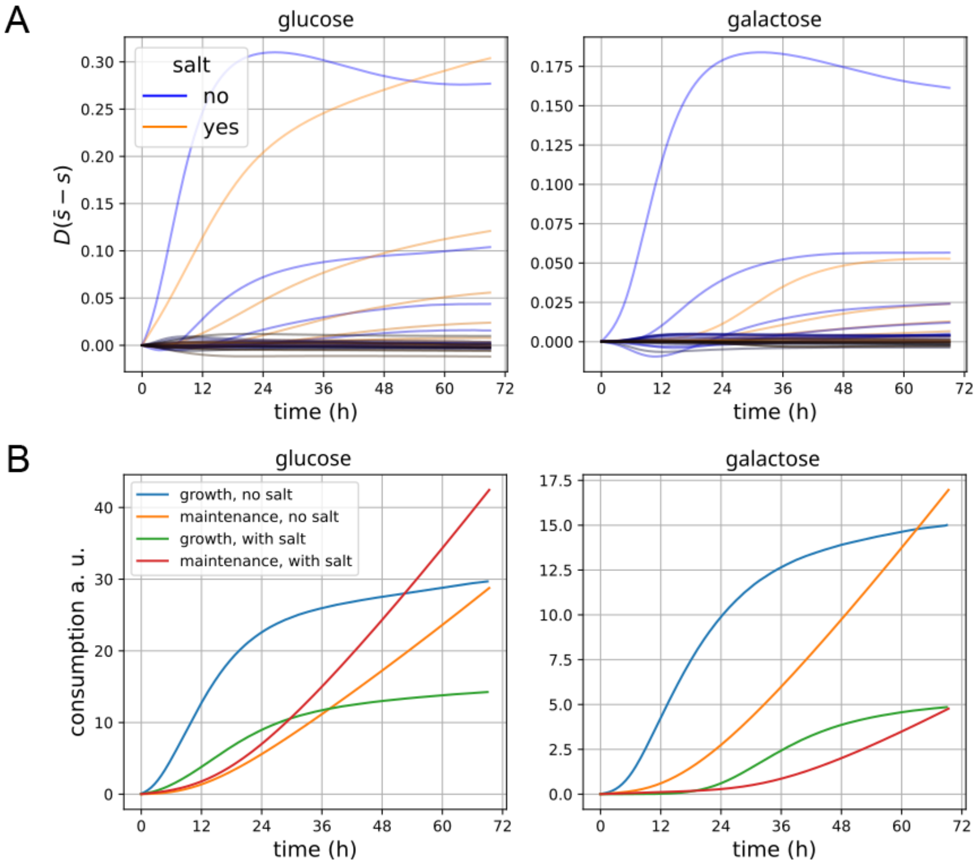

Comparisons between salt-free and salt-containing environments. The growth experiments have been run on two sources of nutrient, glucose and galactose. Additionally, both nutrients have been tested in conjunction with salt or without.

A The nutrient diffusion $D(\bar{s} - s)$ term averaged per layer as in Fig 4A. The 16 layers are for both the salt-free and salt-containing environments represented as a curve getting darker the further the layer is from the grid border.

B The respective contributions to nutrient consumption of population growth and population maintenance, as in Fig 4B. The colours are chosen to group curves according to their consumption term. That is, blue and green for population growth, and orange and red for population maintenance.
