# Supplementary material for: Quantifying massively parallel microbial growth with spatially mediated interactions
Source: PLoS Comput Biol. 2024 Jul 22;20(7):e1011585. doi: 10.1371/journal.pcbi.1011585 (PMC11293690; doi:10.1371/journal.pcbi.1011585)
Supplement: S1 Protocol — (PDF) [file pcbi.1011585.s008.pdf]

S1 Protocol. Pseudo-code for the fitting algorithms.

Fitting the $\alpha_i(t)$

Algorithm 1 Definition of $\alpha_i(t)$, location and time to maximum growth of every colony

function ALPHA(t, r_0, c, m)
    return $r_0 \cdot c/(c + e^{-mt})$

let $J := [0, n_{\text{row}}[\times [0, n_{\text{col}}[$

var t_max : **int**$[n_{\text{row}}, n_{\text{col}}]$
for $i \in J$ **do**
    $\text{t\_max}[i] \leftarrow \underset{t\in[0,n_{\text{t}}[}{\operatorname{argmax}}\, \rho[i,t]$

Algorithm 2 Fitting of the parameters

/* Fitting of the local $\hat{r}_{0,i}$, $\hat{m}_i$ and $\hat{c}_i$ */
var $\hat{r}_0, \hat{m}, \hat{c}$: **float**$[n_{\text{row}}, n_{\text{col}}]$
for $i \in J$ **do**
    $\hat{r}_0[i], \hat{m}[i], \hat{c}[i] \leftarrow \underset{r_0,m,c}{\operatorname{argmin}} \sum_{t\in[0,t_{\max}[i][} \left\| \rho[i,t] - \text{ALPHA}(t, r_0, m, c) \right\|$

/* Fitting of the global r_0 and m parameters, according to the local c_i */
var r_0, m : **float**
$r_0, m \leftarrow$
$$\underset{r_0,m}{\operatorname{argmin}} \sum_{i\in J} \sum_{t\in[0,t_{\max}[i][} \left\| \rho[i,t] - \text{ALPHA}(t, r_0, m, c[i]) \right\|$$

/* Fitting of the local c_i parameters, according to the global r_0 and m */
var c : **float**$[n_{\text{row}}, n_{\text{col}}]$
for $i \in J$ **do**
    $c[i] \leftarrow \underset{c}{\operatorname{argmin}} \sum_{t\in[0,t_{\max}[i][} \left\| \rho[i,t] - \text{ALPHA}(t, r_0, m, c) \right\|$

Algorithm 3 Computation of the $\alpha_i(t)$

```
var alpha : float[n_row, n_col, n_t]
for i ∈ J do
    for t ∈ [0, n_t[ do
        alpha[i, t] ← ALPHA(t, r_0, m, c[i])
```

$$
\begin{array}{l}
\textbf{var}\ \text{alpha} : \textbf{float}[n_{\text{row}}, n_{\text{col}}, n_{\text{t}}] \\
\textbf{for}\ i \in J\ \textbf{do} \\
\quad \textbf{for}\ t \in [0, n_{\text{t}}[\ \textbf{do} \\
\quad\quad \text{alpha}[i, t] \leftarrow \textsc{alpha}(t, r_0, m, c[i])
\end{array}
$$

Fitting the density-dependent model

Algorithm 4 Definition of the k layers of coordinates K_k

$$
\begin{array}{l}
\textbf{var}\ \text{K} : \textbf{int}[n_{\text{row}}, n_{\text{col}}] \\
\textbf{for}\ (u, v) \in J\ \textbf{do} \\
\quad \text{K}[u, v] \leftarrow \\
\quad\quad\quad \min\left(u + 1,\ v + 1,\ n_{\text{row}} - u,\ n_{\text{col}} - v\right) \\
\\
\textbf{let}\ K_k := \left\{ i \in J \,\middle|\, \text{K}[i] = k \right\}
\end{array}
$$

Algorithm 5 Calculation of the nutrient concentrations as φ

$$
\begin{array}{l}
\textbf{function}\ \textsc{phi}(t, k, i, \nu, \alpha, \epsilon, N) \\
\quad \textbf{var}\ \text{sum} : \textbf{float} \\
\quad \text{sum} \leftarrow 0 \\
\quad \textbf{for}\ t' \in [0, t[\ \textbf{do} \\
\quad\quad \text{sum} \leftarrow \text{sum} + \alpha[i, t'] \times \epsilon[k, t'] \times N[i, t'] \\
\quad \textbf{return}\ \exp(-\nu \times \text{sum})
\end{array}
$$

Algorithm 6 Iterative algorithm

/* Initial $\epsilon_k(t)$ values */
var epsilon : **float**$[n_{\text{layers}}, n_{\text{t}}]$
for $t \in [0, n_{\text{t}}[$ **do**
  for $k \in [0, n_{\text{layers}}[$ **do**

$$\text{epsilon}[k,t] \leftarrow \underset{\epsilon}{\text{argmin}} \sum_{i \in K_k} \left\| \rho[i,t] - \text{alpha}[i,t] \times \epsilon \right\|$$

/* Initial value for ν */
var nu : **float**

$$\text{nu} \leftarrow \underset{\nu}{\text{argmin}} \sum_{k \in [0, n_{\text{layers}}[} \sum_{i \in K_k} \sum_{t \in [0, t_{\max}[} \left\| \rho[i,t] - \text{alpha}[i,t] \times \text{epsilon}[k,t] \times \text{PHI}(t, k, i, \nu, \text{alpha}, \text{epsilon}, N) \right\|$$

/* Iterating the fits */
for 20 times **do**
  for $t \in [0, n_{\text{t}}[$ **do**
    for $k \in [0, n_{\text{layers}}[$ **do**

$$\text{epsilon}[k,t] \leftarrow \underset{\epsilon}{\text{argmin}} \sum_{i \in K_k} \left\| \rho[i,t] - \text{alpha}[i,t] \times \epsilon \times \text{PHI}(t, k, i, \text{nu}, \text{alpha}, \text{epsilon}, N) \right\|$$

$$\text{nu} \leftarrow \underset{\nu}{\text{argmin}} \sum_{k \in [0, n_{\text{layers}}[} \sum_{i \in K_k} \sum_{t \in [0, t_{\max}[} \left\| \rho[i,t] - \text{alpha}[i,t] \times \text{epsilon}[k,t] \times \text{PHI}(t, k, i, \nu, \text{alpha}, \text{epsilon}, N) \right\|$$

Fitting the diffusion model

---

function $f(s, K, \kappa)$
    return $\left(1 + e^{-sK}\right)^{-\kappa}$

function $rho(K, \kappa, \alpha, s)$
    return $\alpha \cdot f(s, K, \kappa)$

Algorithm 7 Iterative calculations of nutrient (s) and its averages over neighbours (m)

```
function CALCULATE_S(D, ν1, ν2, K, κ, α, N, m)
    float[n_f, n_c, n_t] s
    float diffusion, consumption1, consumption2

    s[t = 0] ← 1
    for t ∈ [1, n_t[ do
        diffusion ← D (m[t − 1] − s[t − 1])
        consumption1 ← ν1 · α[t − 1] · f(s[t − 1], K, κ) · N[t − 1]
        consumption2 ← ν2 · N[t − 1]

        s[t] ← s[t − 1] + diffusion − consumption1 − consumption2

    return s

function CALCULATE_M(s)
    float[n_r, n_c, n_t] m
    float sum
    int n_added

    for r ∈ [0, n_r[, c ∈ [0, n_c[ do
        sum ← 0
        n_added ← 0
        for i ∈ [r − 1, r + 1], j ∈ [c − 1, c + 1] do
            sum ← sum + s[i, j]
            n_added ← n_added + 1

        m[r, c] ← sum/n_added

    return m
```

Algorithm 8 Iterative calculation of the mean field

```
float[h_r, n_c, n_t] m, s
float previous, current

m ← 1
previous ← ∞
loop
    s ← CALCULATE_S(D, ν_1, ν_2, K, κ, α, N, m)
    current ← LSE(ρ, rho(K, κ, α, s))
    if previous < current then
        break
    else
        m ← CALCULATE_M(s)
        previous ← current

return m
```
