# Supplementary material for: Quantifying massively parallel microbial growth with spatially mediated interactions
Source: PLoS Comput Biol. 2024 Jul 22;20(7):e1011585. doi: 10.1371/journal.pcbi.1011585 (PMC11293690; doi:10.1371/journal.pcbi.1011585)
Supplement: Attachment — Submitted filename: Borse-et-al-reply.pdf [file pcbi.1011585.s010.pdf]

Re: PLOS Computational Biology submission entitled "*Quantifying massively parallel microbial growth with spatially mediated interactions*" by F. Borse *et al.*

Dear Editor,

We would like to thank you and the reviewers for your careful consideration of our manuscript. We are thankful for the constructive comments provided. We have addressed all the issues raised in a revised manuscript and believe that these modifications have further strengthened the work. In particular, the performed additional analyses have clarified the role played by parameter c_i (capturing the colony specific physiological states) to the growth patterns. Furthermore, we have substantially expanded the results section presenting the diffusion model inference. Details of our responses to the comments of the reviewers and the corresponding changes in the manuscript are included below.

We hope that you will find our manuscript, in its revised form, of sufficient quality to merit publication in PLOS Computational Biology.

On behalf of the authors,

Florian Borse and Ville Mustonen

Colour scheme:

Black for structure and for reviewer comments.

Blue for our answers in the reply. An annotated version of the manuscript with main changes highlighted in blue is provided as well.

Reviewer #1: Review summary

Recent experimental advancements have enabled measuring the growth curves of microbial colonies in a massively high-throughput way. However, analyzing such big data with proper correction of noises and biases is still an open problem. This paper disentangled the spatial variability in the previously published "Scan-o-matic" growth assay using machine learning regression models, and further developed explicit mechanistic models with growth profiles and nutrient diffusion that successfully fit the data. Importantly, their computational approaches have great potential to infer various interesting quantities, such as the population variation of lag time, the nutrient uptake rate, and the nutrient diffusivity, from massively parallel growth assays. However, unfortunately, the evaluation of these quantities was not well-conducted in the paper. Instead, they emphasized the importance of spatial resource competition, which was not a very new concept in the field of ecology. Overall, the idea and method presented in the paper are meaningful in analyzing spatial variability in big data generated under a shared environment, but extensive clarification and analysis should be required on what information can be extracted from the data with their model and how reliable it is.

Major comments.

1, The connection to the existing spatial models and the novelty of the paper should be clarified.
At the end of the first paragraph on page 4, the authors claimed “Here, our main aim is to gradually build a mechanistic model to better understand these spatial effects.” However, the final model (“diffusion model”) is not very new by itself, and claiming it as a novelty (as in the 4th paragraph of Discussion) of the paper might leave a wrong impression.

The spatial distribution of nutrients has been modeled in the various contexts of ecology. For example, section 3.2 of the book "Diffusion and ecological problems: Modern perspectives” by Okubo and Levin (Springer) summarized such models in marine ecosystems. In the microbial context, “Bacterial coexistence driven by motility and spatial competition” by Gude et al. (Nature, 2020) described a similar model to this paper. The novelty of this paper should be clarified compared with existing models.

This was not our intention, we agree that resource diffusion has been considered in various models in the literature (and now expand that context in the text in the introduction, as well as discussion). Our main point is that rather than modelling each colony in isolation, a coupling that results from (here latent) resource dynamics turns out to be an extremely efficient, low parametric, model for the whole growth plate. And as a corollary we can see such a high-throughput measurement assay as an interesting system of spatial ecology. This link has not been made before. Similarly, doing an evaluation of the growth parameters of individual colonies, in isolation, would not lead to an unbiased view of the real intrinsic, genotype-specific, growth parameters; this is now highlighted in the discussion and the results section presenting the diffusion model.

To me, the main novelty of the paper seemed the analysis of the various parameters in massively parallel growth assays. The spatial variability in the big data should contain many interesting information that needs to be extracted by a careful computational approach. The main difficulty is that the spatial variability can be originated by various sources: systematic bias (such as temperature gradient), intrinsic stochasticity of cellular processes, and spatial nutrient competition. The method in the paper can disentangle this problem and infer quantities such as the variability of cell’s physiological state (c_i), nutrient uptake rate (ν_1 and ν_2), and nutrient diffusivity (D). The evaluation of these parameters should be presented in the paper. The detailed comments on this point are as follows (in points 2-4).

Thank you, based on your and the other reviewer comments, we agree that in the original submission the non-diffusion related parameters and their variability were not presented sufficiently. We have now performed several extra analyses while addressing the specific comments by you and the other Reviewers (e.g. spatial aspects of physiological state c_i, what are the necessary local parameters, growth on lower colony density plates). In addition, we have considerably extended the results section presenting the diffusion model results e.g. by presenting two new figures.

2, Are fitted parameters realistic and meaningful?
The diffusion model has 1543 dimensions to fit and can get various quantities from the big data. These parameters should be compared with experimental counterparts. For example, is D comparable to the growth limiting nutrient of each plate (plates 1-4)? What is the distribution of c_i? Does c_i have a spatial bias or not? The evaluation of the parameters would be necessary to strengthen the impact of the paper.

We can express the diffusion constant D in standard units dx^2/ds, where ds is the time interval between two images (20 minutes intervals, so 1200s) and dx is the minimal distance between two colonies (the pin pads are of estimated 105.75mm width and 69.75mm dimensions, which if divided by the 32x48 population gives approximately 2.2mm).

We can this way transform the units of our diffusion constants as D * dx^2 / ds (see new Table 1). Our diffusion constants are in reasonable agreement – between 50% to 70% of the diffusion constant of glucose in *pure* water of which is about $7 * 10^{-6}$ [cm^2/s] (Converti *et al.*)

The spatial bias for the c_i can be measured using a random forest model's ability to predict the c_i using location, i.e. $c_i = y(x_i)$. If using again every 4th position as test set, we obtain the following R^2 scores:

R^2	Plate 1	Plate 2	Plate 3	Plate 4
Train set	0.982	0.971	0.752	0.984
Test set	0.936	0.834	-46.161	0.934

We can see that for all plates except plate 3, the location is able to predict the c_i reasonably well (the "test" scores), while for plate 3 the random forest algorithm did not converge to a good fit (the "train" score) in the first place and failed to predict the test set.

This finding should be contrasted to two other predictabilities. The first one is a competing assumption, which would be that the c_i would be driven by initial population size. We can write this model as $c_i = y(N_i(t=0))$, and using the same strategy we obtain the following scores:

R^2	Plate 1	Plate 2	Plate 3	Plate 4
Train set	0.797	0.812	0.808	0.866
Test set	-0.212	-0.502	0.101	0.074

The predictability is here very low for every plate, which discards the idea that the variation in c_i would stem from initial population size.

Another model we can contrast the previous R^2 with is one where we would predict a spatial bias in the initial population size. Using the same approach for a model $N_i(t=0) = y(x_i)$ we obtain the following scores:

R^2	Plate 1	Plate 2	Plate 3	Plate 4
Train set	0.951	0.967	0.961	0.967
Test set	0.675	0.737	0.780	0.797

Here the prediction scores are mediocre despite the random forest algorithm converging to a reasonable fit (the “test” score).

We conclude that both c_i and $N_i(t=0)$ have a degree of a spatial component but $N_i(t=0)$ seems not to be driving c_i variability (this analysis is added to the Results section concerning the diffusion model). We hypothesise that these spatial components reflect some aspect of the pre-culture plate which was shared between the four plates see reply to Reviewer 3 Comment A. We have also systematically considered the local versus global role of the adjustment function parameters (see reply to the next point.)

3, To model alpha_i(t), why was c_i assumed to vary across the positions, and why not r_0 and m? The intrinsic growth term alpha_i models the growth profile of a colony at position i. As alpha_i has three parameters, c_i, m, and r_0, the authors should clarify why r_0 and m were assumed to be constant across the positions. Was the variation of r_0 and m small enough compared to the variation of c_i? Alternatively, can c_i be constant, and instead, can r_0 or m be varied across positions? This point is important because understanding the variability is one of the main aims of the paper (as discussed in the last two paragraphs of page 2). Assuming no spatial variability in r_0 and m needs an additional explanation or analysis.

Thank you, clarifying this point in the revision has been very helpful. Biologically, we believe it is justified that only c is varying per colony as these are isogenic strains. Because cells are genetically identical, they should share intrinsic growth parameters, i.e., maximum growth (r_0) and how quickly cellular states can change (m) (inversely related to lag-time). In contrast, the specific physiological state (c) of a colony is not fixed, because it is largely dictated by the environmental history - which in turn varies between colonies (e.g. they had access to more or less nutrients, depending on position, in the pre-culture. That said, in the spirit of data science, we performed additional analyses which we compared to our chosen model (c local, r_0 and m global). This analysis is now reported in results where we go through the diffusion model. We now as well comment on the important difference between intrinsic and effective growth parameters which are based on local fits in the Discussion section.

Here are the results:

- We fitted all three parameters per colony. There is a small improvement (7.37%), but the resulting model uses 1536*2 - 2 additional parameters per plate, which is a lot of added complexity and goes against the biological basis of the model where r_0 and m are intrinsic growth parameters.
- We fitted global c and m and local r_0, which yields much higher total error and does not converge properly.
- We fitted global c and r_0 and local m, which yields almost no change to total error (0.04% increase).

- We finally fitted global r_0 and local m and c, which yields a very small improvement of 3.67% only. As this increases the number of model parameters (1536 - 1 additional parameters) per plate, we decided that the increase in model complexity compared to marginal decrease in total error is not worth it, especially as it goes against the biological interpretation assigned to the adjustment function model.

Based on these analyses we conclude that the alpha model is flexible enough that we can choose either m or c to be local and get essentially equivalent total fit errors. However, because biologically both r_0 and m can be reasoned to be intrinsic strain specific (here global) parameters, we have kept the original choice of global r_0 and m and local c while adding a statement that local m with c global would also work.

4, Why was f modeled by eq. 17?
The form of f (how nutrient availability impacts the growth rate) was not straightforward to me. For example, Monod growth is a widely-used model, and indeed the cited paper (reference number [40] by Birol et al.) concluded that Monod growth was more appropriate than Teissier growth in their case. Also, the modified version with exponent -\kappa was not familiar, especially given \kappa was very small in the synthetic data set. I would appreciate an additional explanation about the model choice.

Our diffusion model manages to fit all four plates well when using this f function, while in our attempts the more common growth models did not. For example, using Monod growth instead of f results in a poor fit

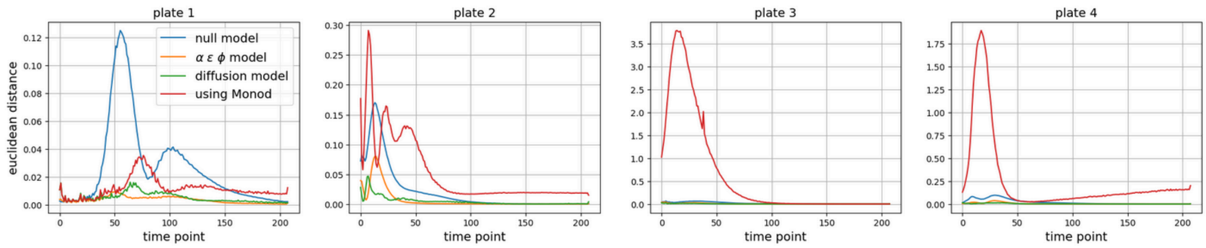

and applying an iterative recalculation of the alpha parameters ends in a divergent process for other than plate 1 data. Please note that for our purpose – demonstrating the ability of a latent resource variable that is coupled via a diffusion process, and a single population specific parameter c_i, as an explanation for the spatial variation in growth – the arbitrary nature of our choice for this function does not impede our reasoning. Does a function f, mapping the population growth to the resource availability, exist when the changes in resource availability are following a diffusion process? We show here the existence of a generic monotonic nonlinear function fulfilling this criterion reasonably well. We also note that the coupling functions f (from nutrients to growth) and F (from cells to nutrient consumption) are both interesting and should be approached in the future in a model-agnostic view guided by the data. To us this goal seems to require measuring both the population and resource dynamics simultaneously (at least we have not made progress keeping both functions open while having not measured resource dynamics). We have expanded this point in the Discussion section and acknowledge the caveat concerning the choice of f and F functions.

Minor comments.

5, Configuration of the "Scan-o-matic" experiment should be clarified in Fig 1 and Method. The graphic layout of the experiments would be helpful in Fig 1 to quickly understand the setup. Also, it might be good to clarify some experimental information in Method, rather than just citing the original paper. Especially, the area of the rim region (outside the grid) and the spatial distance between adjacent grid cites are important as the paper discussed the spatial availability of nutrients.

As to clarify the experimental layout of the input data, we added an image of a plate to Figure 1d. In the methods section, the initial part introducing the data sets now explicitly mentions the physical dimensions of the plates (119mm x 82.5mm) and populations grid (105.75mm x 69.75mm). The distance between populations is approximately 2.2mm, and the rim regions are approximately 6.5mm wide. The methods section now also mentions that the pre-cultures have all been done from the same plate.

6, The assumption of \lambda on page 9 should be explained more.
The explanation about "linearization" of Monod growth was confusing to me. To get \lamda = s from \lamda = s / (K+s), K >> s and K=1 should be assumed. I think the constant K can be absorbed into another parameter, but this should be explicitly explained, especially because s_0 = 1 is used on page 14.

Thank you, based on this and the comments 13 and 14 by Reviewer 2, we conclude that this part was unclearly presented. We have simplified the presentation and removed the \lambda function from the notation. The assumption that K >> s is now explicitly mentioned as well and as the α_i parameter multiplies the whole expression, we in effect absorb the constant K into its r_0 parameter.

Miscellaneous points.

7, When index i is first introduced at the first paragraph in page 2, it should be clarified that i labels the spatial positions. Related to this, the y labels of Fig 1a should be the average of the quantities.

Index *i* labels populations; x_i labels their spatial positions. Changes to the manuscript: Before listing on page 4 the computed population metrics (size, growth and relative growth) the role of index i is introduced by "of the growth of a population i". The labels on the y axis of Figure 1a are changed to be explicit about the averaging, using angle brackets.

8, In the caption of Fig 1, "cultivated for 48 h" should be 72 h.

Thank you, corrected.

9, In Fig 1b and its caption, what "inverse grid distance" means is not clear and should be explained more.
We now use "layer" and explain that in the caption.

10, In the caption of Fig 1c, h of t=72h should be spaced and roman.
Corrected.
11, In the last paragraph on page 10, Figure S1 should be S2. S1 is used for Table and there is no Fig. S1.

Corrected.

Reviewer #2:

I reviewed this paper together with a member of my lab, so this review represents the consensus of our perspectives.

The authors re-analyze an existing dataset of colony growth and identify the forces that drive spatial variation of lag time, growth rate, and biomass yield. First, they apply a statistical approach to explain the variation in terms of spatial position and initial population size. Second, they fit a series of mechanistic models to explain the growth curve of each colony with density-dependent and resource-dependent models of growth. They find that models that capture the decline of resource concentration, together with diffusion, can almost fully explain the spatial variation in growth for the four imaged plates in their dataset.

I appreciated the goal of this research to understand the variation present in these colony growth experiments, which is a frequent challenge for many labs. The technical implementation is solid with mostly good descriptions of the methods (but see comments below for a few places needing improvement), especially the (symbolic) code given by the authors. However, I am unsure about the scientific impact of the paper as written. Although the authors explain the growth rate variation in the specific dataset to an impressive degree, they do not offer general lessons for future experiments or about spatial variation of growth in natural environments. See below for a detailed list of items related to this as well as some more minor issues.

### Major issues

1. I felt like the paper does not provide a clear take-away message for the working theoretician or experimentalist in microbial ecology. The very last paragraph of the Discussion has some thoughts on this issue, but I think the paper would be much stronger if the impact is addressed earlier and throughout the Results especially to better appreciate the significance of the steps. In particular, I still have some questions that I think the authors work can address and would be valuable for the field, but appear to be missing:

We are now emphasising the value that the latent space modelling approach gives in the interpretation of such data and that it is essential to get to genuine intrinsic growth parameters. We have substantially expanded the Results section presenting the diffusion model with new figures added on resource consumption and the diffusion caused variability of the local resource environments.

a. In light of their results, should experimentalists design these experiments differently to reduce variation, or design them in a particular way to accurately account for this information?

If the experiment is performed in order to accurately measure intrinsic growth parameters then our lessons are clear: one should not place, to a shared plate, strains that have large variability in r_0 and the consumption rates as this will inevitably lead to the big differences in the local resource concentrations. To minimise the effect, one can either increase the intercolony distance (trade-off in throughput) or perform a joint global inference of the

intrinsic parameters as done here. If the resource usage profiles of the studied strains differ greatly, it may well be that a single latent dimension is not sufficient to describe the spatial aspect - thus hampering computational approaches to fix the bias. In these cases, other assays, without nutrient diffusion in a shared environment, might be required (and may come at the cost of throughput, as explained in the Introduction). This may concern, in particular, setups with other strong sources of variation except for the spatial layout, such as use of different species or phenotypically very different strains with very distinct growth properties. As we discover a very effective method to control for variation caused by the spatial layout in this study, this physical setup can now be used with minimal unexplained variation for isogenic strains cultured on the same plate. This means that also heterogeneous species or strains can be studied effectively when they are cultured on separate plates. Moreover, we demonstrate the physiological state to be a major source of variation, indeed our only population specific parameter. Thus, better means of preparing samples to reduce variation in pre-culture could be developed. This should reduce variation in, most notably, lag phase length. We have now written about these implications in Discussion and discuss pre-culture optimisation as a possible avenue to remove the final remaining population specific parameter c_i which models the population's physiological state.

b. I didn't find a clear explanation of where the colony growth heterogeneity comes from in the first place. Is it due to initial variation in initial population size or nutrient concentration? Can the authors quantify the relative contributions of those two factors? For example, can the authors simulate data with fewer colonies on the same area, and test if this reduces the spatial variation?

Initial population size variation will create differences in the microenvironments so it is part of the reason. Another reason is the variability in the physiological states of colonies. Both of these aspects have a spatial component, although not in a simple way, where initial population size would predict the physiological state. These are now discussed in the paper with additional analyses that were performed to better understand c_i paratemeter (see Reviewer 1 Comments 2 and 3).

We have now added a scanner image panel of a final time point plate to Figure 1d, which helps to better understand what is driving the spatial aspect. Initial resource concentration is homogenous across the plate but the boundary colonies have more resources available to them because they have less neighbours (new Figure 4a shows the biggest effect of diffusion at the three boundary layers). We also have done simulations on an alternative plate design, with 384 colonies, and show that it would lead to the spatial effects being realised later in the experiment.

The following figures represent the importance of location and population size across time: a) simulated data for 384 colonies per plate and fully global $\alpha(t)$, b) simulated data for 1536 colonies per plate and also fully global $\alpha(t)$, c) simulated data for 384 colonies per plate but local $\alpha_i(t)$ with a population specific physiological state c_i and d) for the original experimental data of 1536 colonies per plate.

a

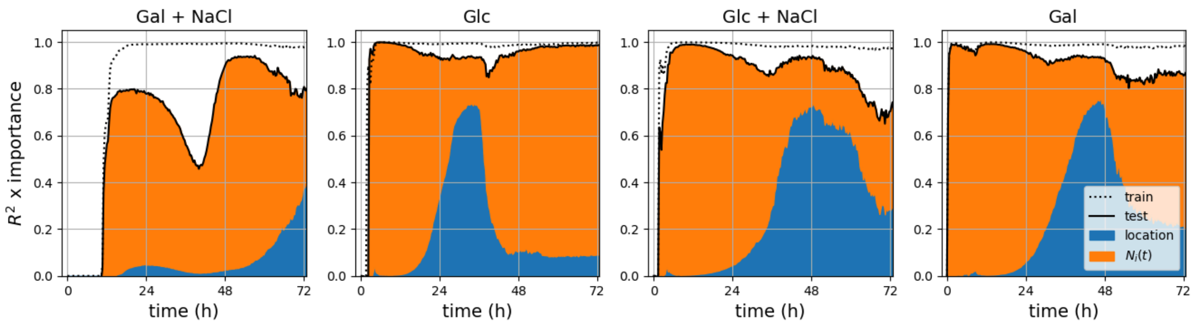

b

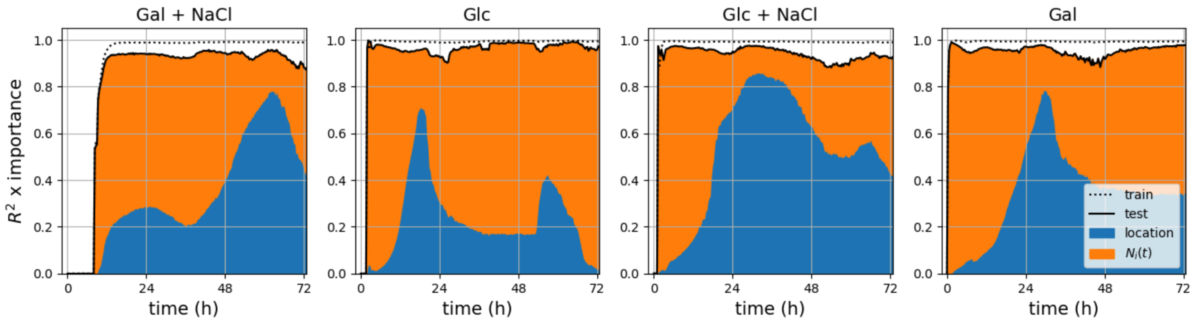

c

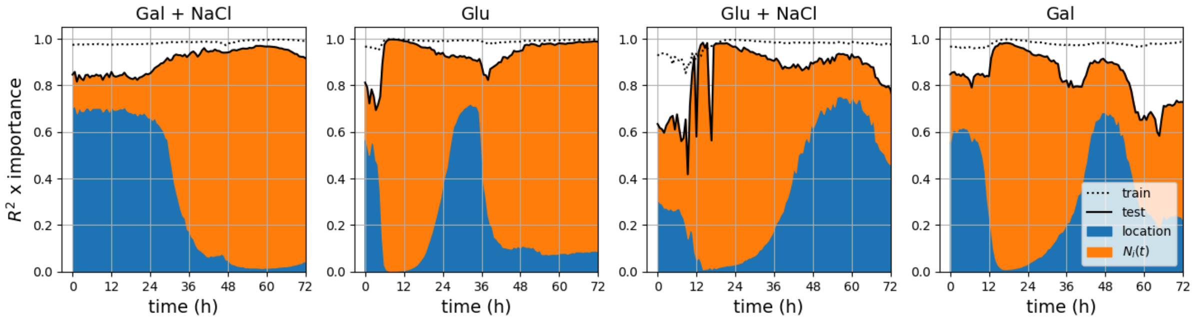

d

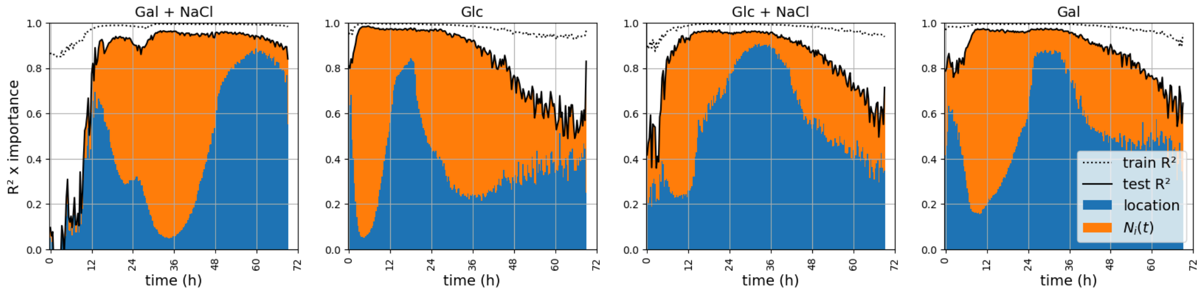

From these figures we conclude that the initial spatial signal is driven by the physiological state c_i (we believe this reflects pre-culture), and the diffusion related spatial signal kicks in later and is delayed when increasing inter colony distances.

Finally, while there is a spatial component in N(t=0) (see the new analysis related to Reviewer 1 Comment 3), it is not driving the biggest spatial signal that is visible in the yields, Figure 2B first panel shows that only plate 1 has a substantial contribution of N(t=0) to yields.

c. What is the relevant scaling between colony distance and diffusion coefficient, between growth rate and diffusion coefficient that are necessary for strong spatial variation in growth rate? This seems valuable to know for assessing the density of colonies on a plate, and how much growth variation that will lead to.

An approximate estimate of the effect can be obtained by multiplying the consumption rate, population growth rate (so how much a population uses the resource per unit time and how quickly it grows) with the diffusion term equilibrating the differences in resource concentrations across the microenvironments. For our data this product nu r_0 D enables us to sort the observed effects in new Figure 4A. The relevant scaling by plating the colonies in a lower density grid is 1/dx^2 (dx being the inter colony distance) – if we plate only ¼ of the colonies the spatial effect should appear later in absolute time, this is indeed what we show (see above reply to your question b). Nevertheless, the spatial effect will become visible also on a low density plate at later stages if the microenvironments differ between locations which is almost inevitable given the boundary regions of the plates. We now highlight in the discussion what things to reflect when designing growth measurements on a shared plate.

2. The relationship between the machine learning model in the first part of the Results, and the mechanistic model in the second part of the Results, are unclear. The flowchart in Fig. 1d suggests that a dependency of the mechanistic model on machine learning model outcome, but they appear to be independent approaches to the same data. Why not just start with the mechanistic model if that provides superior insights?
Was starting with the ML model just intended to be pedagogical, as many readers might intuitively start there as well? Can the authors say if both models are needed or only the mechanistic approach should be considered for similar data in the future? Deciding between statistical and mechanistic approach is a recurring question and deriving such a conclusion here would add to the impact that the article can have.

ML algorithms tend to perform extremely well on various prediction tasks thanks to their ability to handle multivariate non-linear relationships. However, although ML methods are relatively effortless to use in a generic way, they suffer from difficulties in interpretability. Indeed, we use them in most projects as a first step method to generate insight valuable for progress in a project rather than as the final output in our research.

Furthermore, the statistical approach and the mechanistic approach address two fundamentally different problems, namely whether the observed variation in growth between isogenic populations is a “bug” one may want to solve, or a “feature” one can exploit.
Although we ultimately choose the latter, we do start the article with a statistical analysis.

This is motivated partly for pedagogical purposes, but has the advantage of producing a simple model in terms of input features, which combined with a powerful algorithm can give a perspective of how well the data can be modelled with such input features in the first place. As expressed in the article, ML models tend to be too complicated to offer mechanistic understanding of a phenomenon. As the aim of this article is to open the way for new kinds of experiments involving position, that mechanistic understanding is the main means to allow for their interpretation. This in turn explains why this article later focuses on the presented models, with an emphasis on the diffusion of resources. This revised version of the manuscript has added a new layer to the interplay between ML and mechanistic models by

investigating the spatial component of c_i term as well. In summary, we believe that using both ways to look at the data simultaneously is the way forward and our paper shows the power of this “iterative” approach.

3. Looking at the statistical analysis in Fig. 2, I am lacking a null expectation as to how much variation should be explained, for example, by the initial population size and what is "suprising" in the panels of Fig. 2a,b. With the mechanistic model in the second half, the authors have the ability to create synthetic data. What happens, if they apply the same machine-learning random forest pipeline on a synthetically created dataset with logistic growth? Such a plot, potentially in the supplement or as a single panel in Fig. 2, would explicitly provide this null expectation. For the logistic dependence, the increase in variation explained by initial population size (N_0) at the end of the growth cycle seems to be expected, but the earlier rise and dip at the start of the growth cycle no.

We now use the possibility to simulate data to highlight that the initial spatial component of Figure 2A which you refer to stems from the spatial component of c_i (which in turn likely originates from pre-culture). This extra analysis nicely shows the important interplay that one can do between the statistical and mechanistic approaches (new Figure S5, and diffusion model Results section).

If we simulate a data set corresponding to a pure logistical model, i.e. f(s)=s, alpha=r_0 , and set diffusion to 0, ML analysis correctly shows (essentially) no spatial component.

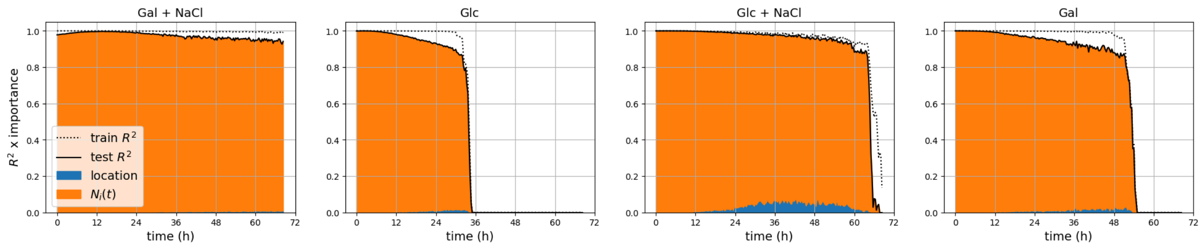

Minor issues

1. In Fig. 1a, second panel: the notation \Delta N for absolute growth rate is misleading, since it suggests a difference in biomass without the units of time. Why not label it \Delta N/\Delta t as they define it in the text?

Corrected according to the suggestion. Additionally, the \Delta N / N shall be in long form (two terms : first the derivative, multiplying the second, 1/N).

2. In Fig. 1a,b, plotting the population abundances on a log scale would help to better identify phases of steady exponential growth by eye. Since the data is already background subtracted, it should be sufficient to simply apply the logarithm to the existing values of N.

We prefer the non log scale visualisations and have kept them in the main text. We have added log scale figures as Figures S6a and S6b.

3. In Fig. 1b, I think it would be clearer to rename the variable "position" as "distance from the border."

Corrected to “layer” which is explained also in the caption.

4. In Fig. 1c, the colors cannot be compared across the 4 plates, because the color scale spans different ranges. Can all of them be normalized to the same range?

Here is a new version of Figure 1c with the data normalised by dividing with the plate maxima, and the colour bars to the same range:

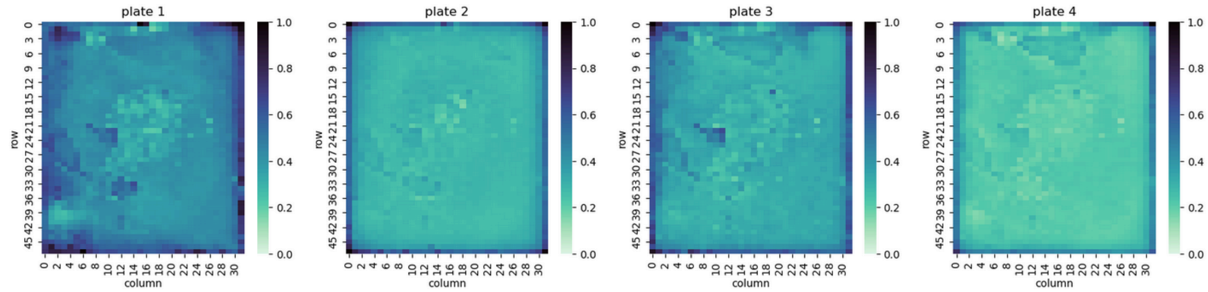

Changes to the manuscript : Figure 1c is replaced with the above figure and the caption adjusted accordingly.

5. So from Fig. 1, we already know that the distance to the border is a good predictor of growth. The machine learning models starts with the raw positions to make a prediction. Can you check if the ML model "rediscovers" the distance to the border as a predictor?

We tried this and the ML model does not perform as well using just the distance, so we have kept both row and column index. Our interpretation is that ML can using both row and column model two effects, one coming from diffusion (which we would expect to be to a large degree captured by the inverse distance) and the physiological state which we show in the revision to have a spatial component, presumably due to the diverging environmental histories of colonies in different positions in the preculture (see Reply to Reviewer 1 comment 2). Indeed, this is the reason why in the ML regression we see the importance of position at the onset of the growth in addition to later stages corresponding to diffusion effects.

6. In Fig. 2, can the authors define explicitly what "importance" means here? The only explanation in the Methods I saw is a technical reference to a scikit-learn variable but without a mathematical definition. Why do we need this weighting factor, and how would the results look without it?

Machine learning models tend to act like black boxes, although when performing a regression task a simple model such as linear regression ($y = f(x_i) = \Sigma_i w_i x_i$) can offer some degree of insight on its use of its input features x_i. That is, by observing how much a feature contributes to the model’s output, which is encoded in the weights w_i associated to each feature.

While random forest algorithms are nonlinear by nature, an analogy to linear regression can be done by using a property of individual decision trees: the ability to measure the impurity of their nodes. A combination of these impurities at a forest level allows to compute for each input feature of a random forest an importance akin to the weights of a linear regression model.

It is important to note that while calculation of impurity is involved during the training of a random forest model, the regression process itself does not use this metric (hence the nonlinear regression despite a linear property). In this sense, the feature importances we use in our ML analysis do not influence the prediction results of any of our models.

As the original manuscript used this analogy in a rather confusing way, we substituted now the term weights with importances, and mentioned in the methods section that these importances are calculated using mean decrease in impurity.

7. In Fig. 2a, can the authors speculate on the variation that remains unexplained? Perhaps there are some stochastic effects inside the cell, e.g., the number of proteins or ribosomes may vary initially between cells.

We think that the test set low performing regions have two different origins, as explained in the main text. The first one likely reflects different initial physiological states of the cell (e.g. nutrient storage status, cell cycle state or protein abundances) so this would genuinely be unpredictable unless the pre-culture strategy can be optimised further (see Reviewer 1 Comment 3 and the identified spatial component of c_i, and the related new analyses).

The other major aspect is the very small growth rates in those regions - meaning that the signal to noise ratio is getting smaller, and as a consequence the signal becomes unpredictable.

8. In Fig. 2b, the plates with NaCl (plates 1 and 3) seem to have much less of their growth rate explained than the plates without NaCl (plates 2 and 4). Is there any plausible reason for this?

Thank you, we did not spot this before as the total per plate errors and their spatial distributions look very similar across all conditions, (see S1 Table and S2 Figure). This means that the full modelling of the growth rates functions rho(t) does not have such an effect even when using the ML approach.

But, indeed, ρ_{max} (as directly extracted per colony) under the ML analysis (Figure 2b middle panel) appears to have a trend. However, we have to be careful with the per colony inferred parameters in the ML part as the r_0, the way we understand it in the mechanistic model part, is an intrinsic, strain specific, parameter and should biologically be a global constant (see reply to Reviewer1 Q3). We speculate that if the NaCl condition would introduce more variability to the start of the growth (in effect via c_i and m parameters) then this would reflect when a given colony would hit maximum growth ρ_{max} . If this were to happen much later than the colony's neighbours it would possibly lead to the realised maximum to be lower than the real intrinsic r_0, thus introducing lack of predictability when done in the colony specific

analysis using ML. We can speculate on the role of NaCl a bit more. In absence of stressful levels of NaCl, colony growth is primarily limited by the local access to usable carbon (all nutrients being present in excess, while usable carbon is initially set to a growth (yield) limiting concentration of 2%). But in the presence of stressful levels of NaCl, colony growth is to a substantial degree affected by the local concentrations of NaCl. NaCl reduces growth through two separate effects. First, the Na+ ions are toxic intracellularly, with in particular the distortion of the Na+/K+ balance reducing growth. Second, the NaCl serves as osmolyte extracellularly, lowering the external water potential and causing water to leave the yeast cells. Yeast cells thereby lose volume, and begin to suffer from macromolecular crowding. At high NaCl concentrations, the cellular countermeasures to Na^+ and osmotic stress (primarily: increased efflux of sodium and internal production of the osmolyte glycerol), are insufficient or too costly for cells to maintain an optimal growth rate, and suffer from both Na^+ and osmotic stress. If either one of these effects increases the effective variability in c_i that would likely result in the relative lack of predictability in ρ_{max} as inferred for each colony locally. As this observation is based on the local effective rates we have not expanded on it in the text where the focus is on intrinsic growth and the power of our approach to explain the variability in the effective rates.

9. In Fig. 2b middle panel, we see that the initial population size can explain some of the variation in maximum realized growth rate. However, what is the directionality here? Do larger populations grow faster?

We can assess this directionality by calculating directly a correlation between these parameters. In our case, we compute the Pearson correlation coefficient between the initial population size $N_i(t=0)$ and the local extracted growth rate ρ_{max}:

Plate	$N_i(t=0) \sim \rho_{max}$
plate 1	-0.2430
plate 2	-0.9205
plate 3	-0.5347
plate 4	-0.8651

As can be seen, larger initial colonies have a lower maximum realized growth rate, this was noticed also in the original Scan-o-matic paper. Again, we emphasize here that working with the “effective” or “realised” values as opposed to intrinsic (our global r_0) makes the interpretation of the growth rates a challenge as the variability in the physiological states and the ensuing differences in local resource environments modify the realised values. For this reason we have not included this analysis to the text.

10. For the comparison in Fig. 3: There are established methods to compare hierarchical models, such as AIC or BIC that also take into account the number of parameters. Did the authors consider using such a criterion but then discard it? It would be interesting to understand the reasons.

Thank you, we have now added AIC analysis. While we are not explicitly doing probabilistic likelihood-based scoring, we can apply AIC to our results by using the variance in our errors as maximum likelihood estimate (Burnham and Anderson), which leads thus to 2k + n * ln(SSE / n) where in our case n = n_rows * n_columns * n_timepoints. Here are the null model subtracted scores for every model and plate (smaller values indicate better models):

Model	k	plate 1	plate 2	plate 3	plate 4
null model	208				
$\alpha\varepsilon$	1756	-2.33×10^5	-4.91×10^4	-3.51×10^4	-9.05×10^4
$\alpha\varepsilon_k$	5026	-3.39×10^5	-2.34×10^5	-8.25×10^4	-2.61×10^5
$\alpha\varepsilon_k s$	5027	-6.04×10^5	-3.65×10^5	-2.99×10^5	-4.36×10^5
diffusion	1543	-5.21×10^5	-4.82×10^5	-4.66×10^5	-5.85×10^5

The results corroborate our presentation: except for plate 1, every model yields a better score compared with its predecessor. In the case of plate 1, the diffusion model obtains a lower score than the $\alpha\varepsilon_k s$ model, due to its lower goodness of fit, but it would still be to us less biologically valuable with the black box of $\varepsilon_k(t)$ modelling the spatial effect. We believe that these AIC scores can be of value to the manuscript and have added them to Table S1.

Burnham KP, Anderson DR, editors. Model Selection and Multimodel Inference. Springer New York; 2004. doi:10.1007/b97636

11. Figure 2 nicely distinguishes between the model fit to test and train subsets of the data, but I did not understand if the authors did a similar cross-validation for their mechanistic models in Fig. 3. Can you please clarify?

For non machine learning models we use all the data to make the fits. This is necessary as the ML analysis shows that there are some genuinely local parameters (c_i) that we believe are fittable but not predictable. This is another example of the interplay between the statistical ML type and mechanistics models where we can lift insight from ML to the mechanistic modelling. We have further performed a test of the mechanistic diffusion model by generating a synthetic data set using the model with the parameters inferred from real data. Re-analysing the synthetic data using our inference method very closely recovers the input parameters, thus supporting the veracity of the implementation as well as self-consistency of the approach (Figure S5).

12. The authors use the term "model free" to describe a statistical model that uses a separate parameter \epsilon(x,t) for every cell and time point. However, this is still a model in the terminology of linear regression, unless I misunderstand what the authors meant. Perhaps rephrase as "mechanism-free" or "random-effects model"?

Thank you, we fully agree and very much like the term “mechanism-free” and have adopted it, as it better describes what we do.

13. After Eq. 5, the authors define a form of choice of phi as the function lambda, which they refer to as a Monod-type model. I found this text and the accompanying section in the Methods (Eq. 11) too mysterious --- why not explicitly write out phi as the Monod model in the main text here, which will be familiar to most readers? Or just write the linearized approximation that the authors actually use. It seems unnecessarily complicated to have separate notation for phi and lambda when they only consider this one example.

Thank you, indeed this part was presented in a rather convoluted way and we have now removed the superfluous symbols and simplified this part. (See also Reviewer 1 , comment 6). This means the model $\alpha\varepsilon_k$phi is renamed as $\alpha\varepsilon_k$s.

14. I think I am missing something in the derivation of Eq. 6 It appears to come from integrating Eq. 5 with dN/dt replaced by Eq. 4 times N. Maybe I am being naive, but why can't one directly integrate Eq. 5 (irrespective of the form of dN/dt) to obtain the proportionality s = nu * N + constant?

Thank you, this is an important point that we have now streamlined the presentation of here (see previous answer as well). While we use the state variable N as given, we are not using dN/dt as evaluated from the data anywhere other than when computing the fit errors. So we are modelling dN/dt (or dN/dt/N to be precise): that is the reason why we need to insert the model of dN/dt to eq 5 before integrating to get s(t) which dependents explicitly on N and its time integral, but not on dN/dt. This is in fact how a symbolic regression approach would work (see Discussion). The goal is to find an analytic expression to a response variable here dN/dt/N as a function of some state variables, here N and *latent* s. We have now simplified the text around Eqs. 4 to 6 to be explicit about this. One could, alternatively, seek directly a form for relative growth rate \rho(t) which would be only a function of the instantaneous population size N(t). For the case of a simple logistic growth equation (see below), this would be possible. However, a formulation that goes via an explicit resource variable (albeit a latent one that couples to the whole N(t) up to time t, Eq. 6) allows for a mechanistic interpretation of the reasons behind the density dependence, and indeed the spatial modelling that we do later.

I would also appreciate if the authors could explain the connection of this approximation to logistic growth --- in the simplest case, this is what would come out for dN/dt when approximating the Monod dependence as linear in the resource concentration and substituting this back into the growth equation for dN/dt (i.e., dN/dt = r * N(K - N)).

The connection to logistic growth can be seen via the coupled equations:

dN/dt = r N s
ds/dt = -nu dN/dt

Identifying:

M = s + nu N → dM/dt = ds/dt + nu dN/dt = -nu dN/dt + nu dN/dt = 0

That is, M is a constant of motion and allows us to separate the equations:

dN/dt = r N/M (1 - nu N/M), which is the logistic equation with a carrying capacity M/nu, as M is constant we have M = s(0) + nu N(0). Similarly we get an equation for s: ds/dt = -r (M-s) s.

Therefore in the special case where both \epsilon_k(t) and \alpha_i(t) are constant the model would be the standard logistic growth model (we state this link now in the text).

15. The first paragraph of the Discussion uses some mysterious-sounding language ("shared environment," "experimental units," "false narratives"). Can the authors reword this using more straightforward language? I think the authors are criticizing the tendency of other studies to dismiss variation as some incomprehensible noise while they show that this variation is quite comprehensible given adequate models, but I think they can state this more explicitly.

Thank you, we have simplified the language.

16. Also in the Discussion section: the term "common garden" has a specific meaning in ecology, but this is not what the authors seem to want to invoke here. Compare to the use in https://doi.org/10.1038/hdy.2015.93 and rephrase if necessary.

Thank you, our usage was not referring to the specific ecological term, we have removed it.

17. In the Methods section: Can the authors explain further what reasons made them choose the functional form in Eq. 17? In particular, the authors should explain why they use this model rather than more common models (mainly Monod, or a few alternatives like Droop, Blackman, Bertalanffy, etc.) and what the consequence of that choice is. This should also be addressed in the main text around Eq. 9. The related sentence in the Discussion also did not make sense to me: "We simply opted for a generic form of a monotonically decreasing nonlinear function – a generic form can be justified, as growth characteristics can exhibit different modes, including the uncoupling from nutrient availability [42]."

Please see our reply to Reviewer 1 question 4. In short, using a Monod function did not work. We have moved Fink et al. to the place where we discuss growth variability and its impact on experimental design.

Reviewer #3

In this manuscript, the authors study the origin of spatially heterogeneous growth observed in massively parallel microbial colony assays. They follow a dual approach in which they first use a random forest machine learning approach to assess the relative importance of colony location and size for the prediction of relative growth rates under different conditions. Finding temporally varying contributions, they proceed to construct a series of mathematical models that gradually introduce more intricate couplings between population growth and the local environment to unravel the underlying dynamics.

In general, the paper is well-written and easy to follow. The approach and findings are well presented and of potentially significant interest to the wider scientific community. The scan-o-matic platform used to generate the experimental data in this manuscript belongs to a class of massively parallel growth assays that is widely used in the yeast genetics community and beyond. Positional artifacts are an inherent challenge of such assays and non-trivial to compensate for. While the isogenic implementation of the assay used in this work does not reflect the complexity of a typical assay, featuring a multitude of different

genotypes and growth rates, it serves as a well-controlled test bed for the machine-learning-informed modeling approach.

Given the rapid rise of machine learning application in science, understanding how it can inform the design of interpretable mathematical models in complex (biological) systems is of major importance, not only to life sciences but to the scientific community in general. The well-controlled yeast-based model system investigated here is ideal to demonstrate the conceptual feasibility of the approach and showcase how key features of the observed growth dynamics can be rationalized in a meaningful way without detailed insights into the underlying mechanisms of colony expansion.

The authors claim to have shown how the coupling of population growth to their local environment and the interactions between environments can explain the observed spatial growth patterns. In addition, they claim to unravel how the temporal variations in relative importance of space and population size are coupled to different growth phases of the population. Both findings are supported by the presented data and analytical models. While the impact of resource diffusion on spatially structured growth may be of limited novelty, I find the presented machine learning approach conceptually relevant and the ensuing insights on temporally varying contributions of intra- and interpopulation insightful and important.

Major Comments

Comment A.
The experimental data presented in Fig. 1c shows very distinct identical patterns across all four plates (see e.g., areas around column 6-12, row 21-27 or column 10-22, row 2-9). Given that each of the four plates supposedly represent independent experiments, these patterns imply the presence of either experimental or data preprocessing artifacts. Please investigate and explain the origin of these patterns and correct your analysis pipeline accordingly. In addition, please provide access to the unprocessed raw data (currently missing).

Apologies for the missing link to the raw data, we have added the raw data to GitHub and also linked to the original Scan-o-matic GitHub repository where also the raw images of the plates can be found.

For the artefacts (thank you for highlighting them), in short we believe their origin is in the pre-culture that was shared between all four plates. We added text to the corresponding figure caption and Methods section about this effect. Here is a more nuanced explanation of how the effect could have arisen. Complete independence of experiments is not possible - all factors affecting growth cannot be completely randomised. We believe that remnants of such dependency, rather than any pre-processing artefacts, create the observed shared patterns. In particular, the four experiments were generated from the same pre-culture plate. They will therefore share some growth features that vary depending on the properties of pre-culture plates. The shared features may encompass multiple colonies in a row or column wise fashion, and then typically derive from a systematic effect on initial population size. The initial population size is a function of 1) how many cells are picked up from the pre-culture plate, by each pin on the plastic transfer pads, 2) and the fraction of those that are picked up that are deposited in each colony position on the experimental plate. The latter depends on

not only random factors, but also on systematic factors, such as the structure of the medium surface of the experimental preculture plate, which rarely is perfectly even. In the same way, the number of cells that are picked up by pinheads from the preculture plate also depends on the structure of the medium surface on the preculture plate. If the medium is not perfectly even, for example due to adhesion between the nutrient medium and the walls of the plastic plate itself such that the medium surface slopes slightly close to plastic walls, the rigid pin pad will tend to pick up fewer cells from central colonies than from peripheral colonies. This then, in turn, leads to a lower initial population size for central colonies on experimental plates. The bias from the preculture plate can also be colony-specific, for example if a bubble or small crack in the nutrient medium surface close to a colony position on the preculture plate interferes with growth of that colony. This may lead to the corresponding pin on the pin pad missing picking up very few or no cells from the preculture, and consequently to no or very noisy growth for the corresponding colony position on all experimental plates. We also note that there is a general dimension to preculture effects, as the amount of nutrients/energy that preculture colonies can access will vary systematically across positions in the same way as for colonies on the glucose experimental plates. This will affect e.g. the amount of nutrients/energy that is stored in precultured plates, and thus the physiological state of preculture colonies at the time of transfer to experimental plates. Finally, we cannot exclude an influence from other dependencies, such as use of plastic plates and pin pads from the same production batch and the experimental plates being analysed in the same instrument, the same fixture to keep plates fixed and the same calibration strip to account for variations in light conditions. Optimising the experimental design and analysis pipeline to account for such effects, by standardisation, randomization and posterior error correction is something that we are continuously working on.

Finally, in the revised version we highlight that our best model has only a single population specific parameter, c_i modelling the physiological state. We believe this is rather impressive and see as the only way to "remove" this local non-intrinsic parameter would be to further optimise pre-culture protocols.

This is of especially high importance since only one measurement per condition is given. Even though many colonies are imaged per time point, the entire ensemble of colonies only represents one data point per image (e.g. when calculating the importance of location) . If at all possible, including more technical replicates would greatly strengthen the statistical analysis of the presented claims. If this is not feasible, explain why and discuss the implications of these statistical limitations.

Thank you, for this data set this is not possible as the original experiment in a way had the replication done in the plate by measuring 1536 times the same genotype. The platform has been used by high replication within a plate and using every fourth colony as a spatial control. Going forward when planning spatial experiments as envisioned in the discussion, replication of whole plates clearly will become important, and we have written this now in the discussion. *"In such experiments, each plate would correspond to a single replicate of the spatial ecosystem so one would need to measure several independent plates".*

Comment B.

In the current structure of the manuscript, showing how spatial variability in growth can be attributed to couplings between population growth and local environments is presented as

the main finding. Given the limited novelty of these insights (see e.g. refs. [1,2] below), I suggest that the authors reconsider the hierarchy of their claims, giving priority to their more interesting findings of temporal variations in location importance and the concept of their dual approach.

Thank you, we acknowledge that we had overlooked the study by Chacón et al. demonstrating how neighbouring colonies affect each other's growth through the diffusion of limiting nutrients. We consider this to represent evidence supporting the biological interpretation and viability of our discoveries, and now cite this work. We now also mention this explicitly in the Discussion (start of page 14). We have further emphasised the power of the dual approach, which has strengthened in the revision as we obtained a more nuanced picture of the final local parameter $c_{i,}$ and linked it to pre-culture.

Comment C.

Throughout the manuscript, the authors several times allude to a biological context which they promise to discuss later. However, it was unclear to which part of the manuscript this referred to. It would be helpful if the authors could clearly indicate where this discussion can be found. Such a more thorough discussion of biological implications and potentially explanations for the phenomena observed in the data, such as different population growth phases, would greatly increase the relevance of the work for a more biological audience. For example, the exponential phase, mentioned several times, should imply a constant growth rate, yet no area of constant growth rate can be observed in Figure 1a.

Thank you, we have now fixed the two "discussed later" points which were not clear or helpful by removing them. We now have an extensive discussion about biological aspects when we dissect the results of our best model, i.e. the fully mechanistic diffusion model.

Figure 1a panel which shows the relative growth rate should be a constant as a function of time when the colonies are growing exponentially. For each plate there is a short window where this is the case, however, due to the variability of the initial physiological states and local environments averaging blunts that region of constant growth which for individual colonies is also relatively short.

Comment D.

Figure 4 is only briefly explained in one sentence in the main text and the figure caption. It would be beneficial if the authors could further elaborate on additional results from this figure and the conclusions drawn from them. Can you link the different behavior observed in the four environments to the underlying biology, such as different lag times, mode of colony expansion (exponential vs. boundary driven growth), etc.? A clear rationale for how different types of environments contribute to the overall findings (beyond demonstrating the robustness of the approach) would greatly help to motivate the design of the study.

Thank you, we agree that the discussion of Figure 4 was not helpful, and have now removed it. We now report the diffusion model results much more extensively by starting with the inferred parameters (Table 1) and revealing the interpretation of the latent resource space: spatial effect, new Figure 4a and resource usage new Figure 4b.

Minor Comments

Comment E.
The introduction would benefit from a more thorough discussion of why interpretable models are so crucial, especially in the context of current challenges in explainable AI.

The main point is that while it is relatively straightforward to predict various variables via ML many key scientific problems of our time are not about prediction only. One example is the idea of systematic control of the growth of microbial populations (applications in antimicrobial therapy as well as biotechnology), in which we need a mechanistic basis for optimising over the space of control protocols that each will lead to some growth behaviour. As far we can tell, this challenge is not convertible to a set of ML tasks focusing on prediction. We have added this point to the introduction.

Comment F.
Please include a more detailed description of the experimental assay. An example of how the plate with colonies used in this experiment looks like (e.g., as part of Figure 1) would be ideal. Explain how N(t) is calculated. Is it a colony's area or the estimated number of cells (taking into account the vertical dimension of yeast colonies)?

We now state here that there is a calibration function that was developed as a part of the platform to achieve such absolute numbers and refer to the Scan-o-matic for the details of how images are converted to N(t). We also added a new panel to Figure 1 showing one plate image at the end of the experiment at 72h.

Comment G.
The order of plates seems arbitrary and confusing. Why this order? Ordering plate numbers with respect to growth speed and or conditions (e.g., gluc, gal, gluc+NaCl, gal+NaCl) would help the reader to keep track of conditions. Maybe even better: Substitute all plate references with the descriptive names while still using the aforementioned order in figures layouts

Changes to the manuscript: As suggested, the plates are labelled according to their medium instead of an arbitrary number (we have kept the order of plates). Figures now use either abbreviated forms (Gal, Glc, NaCl), and the manuscript refers to salinity (salt-containing/salt-free) when plates 1 and 3 are grouped.

Comment H.
Units of actual time (min or h) for the x axis in Fig.1 would be more informative than time steps.

Corrected according to the suggestion extended also to Figure 2cd, and Figures S2 and S3. Additionally, time points mentioned in the paper can also be translated into concrete time units.

Comment I.
Explicitly explain how “importance” in Figure 2a is calculated. Given the prominence of this value for the message of the paper, merely pointing to the documentation of scikit-learn seems too vague.

See Reviewer 2, minor issue 6, for the changes in the manuscript.

In short, scikit-learn calculates the importances for a random forest using the mean decrease in impurity method, which involves calculating the impurities of the nodes in the forest’s decision trees, and comparing these values as to find out which splits have the greatest effect, which can be combined across the forest into importances.

Comment J.
Explain how Euclidian distance is calculated and why it is chosen as the error metrics in Figures 3 c and d.

The term “euclidean distance” was actually misleading, as we are not applying the square root to the distances, and the manuscript should now reflect that. In practice, what we are calculating corresponds to the sum of squared errors (SSE). This is now applied in the manuscript and figures.

These distances (fit errors) are calculated the following way in Figure 3: for every model, we take the distance (value-by-value differences squared, and then summed) between its predictions \hat$\rho_i$(t) and the ρ_i(t) calculated from the experimental data.
The way the figures a & b differ from figures c & d are by how the various populations *i* and times *t* are grouped in order to measure these distances (fit errors) : figures a & b differentiate between populations *i* and hence sum for a given *i* the (\hat$\rho_i$(t) - ρ_i(t))² over all *t* , while figures c & d differentiate between times *t* hence sum for a given *t* (sum for a given *t* the (\hat$\rho_i$(t) - ρ_i(t))² over all the populations *i.*
This is a standard error metric and is used for its ease of computation and interpretability, as well as its punitiveness for greater differences.

Comment K.
Explain the parameter K in Figure 4.

We have removed the old Figure 4 from the paper (both K and kappa are shape parameters which on their own are not that interesting biologically) and present a new figure 4 focusing on the resource dynamics.

Comment L.
On the right hand side of Equation (7) the derivative should be partial.

Thank you. Changes to the manuscript: The derivative “ds(x_i, t) / dt” in the manuscript is replaced with a partial derivative “\partial s(x_i, t) / \partial t”.

Comment M.

A main focus of the manuscript is the coupling of population size/growth to the local environment and the interaction between neighboring environments (see e.g. the third paragraph on page 12: “Thus, variability in growth across the populations of a plate is accounted for by the interactions between neighbouring populations resulting from this diffusion process.”). At the same time, the authors are clearly aware of the importance of boundary effects (in the same paragraph as above: “Due to the physical construction and layout of the plate, populations are exposed to a larger flow of nutrients and energy sources the further out in the grid they are located, which explains the variability pattern in growth across a plate.”). Yet, an explanation of how the two are linked (presumably via boundary effects on nutrient diffusion) is missing.

Naively, I would assume that boundary effects and initial nutrient consumption are the cause of the overall spatial gradient which is then dynamically modified by local consumer-resource dynamics, correct? Is the feedback on these local dynamics of positive or negative nature?

Yes, based on our research we believe that the boundary padding of the plates are causing the main spatial signal and are creating a spatial gradient of resources like you state. We have visualised the per layer averaged D(\bar s_i - s_i)(t) from the best models to show this effect in new Figure 4a for all plates.

References

[1] Chacón, Jeremy M., Wolfram Möbius, and William R. Harcombe. "The spatial and metabolic basis of colony size variation." The ISME journal 12.3 (2018): 669-680.
[2] Baryshnikova, Anastasia, et al. "Quantitative analysis of fitness and genetic interactions in yeast on a genome scale." Nature methods 7.12 (2010): 1017-1024.
