# Supplementary material for: Quantifying massively parallel microbial growth with spatially mediated interactions
Source: PLoS Comput Biol. 2024 Jul 22;20(7):e1011585. doi: 10.1371/journal.pcbi.1011585 (PMC11293690; doi:10.1371/journal.pcbi.1011585)
Supplement: Attachment — Submitted filename: Borse-et-al-reply.pdf [file pcbi.1011585.s011.pdf]

Re: PLOS Computational Biology submission entitled "*Quantifying massively parallel microbial growth with spatially mediated interactions*" by F. Borse *et al.*

Dear Editor,

We would like to thank you and the reviewers for carefully evaluating our revised manuscript. We are happy to see that the overall response to our extra analyses is positive. We have addressed the remaining comments in a second revision. Details of our responses to the comments of the reviewers and the corresponding changes in the manuscript are included below.

We hope that you will find our manuscript, in its revised form, of sufficient quality to merit publication in PLOS Computational Biology.

On behalf of the authors,

Florian Borse and Ville Mustonen

Colour scheme:

Black for structure and for reviewer comments.

Blue for our answers in the reply. An annotated version of the manuscript with main changes highlighted in blue is provided as well.

Reviewer #1

Overall, the authors have clearly addressed the review comments and presented the extended analyses that strengthened the significance of the paper. The authors completely changed figure 4. The new figure 4 nicely shows the detailed behavior of D and F in the diffusion model.

Thank you.

However, some concerns about f(s), which was shown in the previous figure 4, were not clearly discussed.

Major comment.

1) I would appreciate an elaboration on the biological implication of f(s) function (equation 10). All the reviewers had comments on this (Reviewer 1 Comment 4, Reviewer 2 Comment 17, and Review 3 Comments D and K), but the authors did not provide a plausible answer.
In the response to Reviewer 1's Comment 17, the authors stated that the main purpose of the diffusion model was to demonstrate the ability of a latent variable s, and the function could be arbitrary. In the response to Reviewer 3's Comment K, the authors wrote that K and \kappa (shape parameters of f(s)) were not that biologically interesting.
However, a reader would think whether the fitted f(s) can give biological information or is biologically realistic. The very small values of exponent \kappa in Table 1 seemed weird, and I would appreciate a further discussion. In what situation was the form of f(s) derived in the original context? What does the small exponent \kappa imply? To discuss this, it may be helpful to show the previous figure 4 (about the shape of f(s)) in the SI.
There could be multiple possible combinations of the form of f and F, as written in the sentences starting from "As a caveat". However, a biological insight could support a specific choice of f and F. I appreciate the new figure 4, which shows the detailed behavior of the model. I would also appreciate if the authors could add a discussion about the shape of f(s).

Thank you, upon reflection, we agree that removing the original submission Figure 4 was not helpful. We have reintroduced a figure showing the actual f(s) functions with the inferred parameters as a main text Figure 5. We believe these shapes represent biology in the most direct way, not the parameters themselves. This was the point we tried to convey in the reply to reviewer comments last time. In particular, \kappa and K have some fitting flexibility in that one can keep the biology, i.e. f(s) shape, almost invariant while changing both, \kappa and K simultaneously to a degree; this effect is visible from SI Figure 4.

We have added to the results section concerning the diffusion model: *"Shape parameters K and κ of the function $f(s)$, which maps the resource concentration to growth, are in themselves somewhat hard to interpret. However, the biology encoded by these parameters is directly observable by plotting the functions. Figure 5 shows that the concentration to growth functions in galactose conditions decrease faster as resource concentration get lower than those in glucose."*

Minor comments.

2) I appreciate the extensive analyses about whether c, m, and r_0 should be local or global. The additional analyses showed the flexibility of the approach and strengthened the paper. However, it might be better to re-write the argument about the parameters.
It is not trivial that r_0 and m are global, because both should depend on the environment (as discussed in the original Baranyi and Roberts paper), which is likely to be spatially heterogeneous in the case of the Scan-o-matic experiments. I think that the spatial dependence of r_0 is extracted as \epslion_k, and r_0 may be global by definition. m can be possibly local, but the environment should be spatially uniform at the early time before colonies grow and compete for nutrients, supporting the argument that m should be initially global. Just relying on the "biological interpretation" of m to argue m~global might be misleading.

We share your interpretation of the way that the epsilons extract the later (local) variations for our global alpha parameters, and the manuscript has been modified to include a justification for the choice of keeping r_0 and m global (earlier growth phases), and an explicit mention that the $\varepsilon_k(t)$ aim to capture their later variations.

Added a sentence: *"This is justified as in the initial phase of the growth experiments, populations do not compete for nutrients yet, hence their environment is sufficiently homogeneous to consider r_0 and m global."*

Extended a sentence: *"We utilise this information to constrain a location-specific mechanism-free parameter $\epsilon(x_i, t)$ to k layers of locations equidistant to their closest grid border* ***which aims at capturing the biases induced by variably growing populations in the later phases of an experiment****"*

(This is just a comment and not a suggestion) The additional analyses showed that r_0 was global, but m could be local. As m and c_i are both related to lag time, it seems that a simpler model with a single parameter for lag time (instead of two parameters c_i and m), such as the model by Hills and Wright (1994), may also fit the data.

Thank you for the comment. For the present study the question is not about model complexity *per se* as replacing m and c_i by lag_i would in essence leave the number of parameters the same (removing a single global parameter) rather whether the direct lag formulation would make the results more interpretable. We have so far appreciated the straightforward interpretability of the adjustment function formalism as presented by Baranyi and Roberts but will investigate Hills and Wright 1994 in future projects around growth.

3,In Figures 4a and 4b, it might be helpful to have a plot (potentially in the SI) in which the four environments (or two, a sugar type with and without salt) can be compared in a single y-scale. It is especially important for figure 4b as the authors discussed the difference between the conditions with and without salt in the section "Explicit model of colony, environment and inter-environment interactions" (in the paragraph starting from "The comparison of both consumption").

Comparing consumption of the same nutrient under different conditions is indeed relevant. We created two figures S7a and S7b as suggested, in which we display modified versions of figures 4a and 4b, while grouping similar environments into respective figures.

4, Related to Reviewer 3 Comment G. I appreciate the descriptive names for the different plates, but the order seems confusing. They should be ordered based on the sugar type or the existence/absence of the salt. A minimal change from the current order could be swapping Glc and Gal to make “Gal + NaCl, Gal, Glc + NaCl, Glc”, but another order is possible.

We have reordered them by the suggested principle albeit in reverse order. That is, by environment most favourable to growth (Glc) to least favourable (Gal + NaCl), but keeping the presence of NaCl consistent as suggested.

Miscellaneous comments.

5,
When “mean field” is used as adjective, it may be better to write “mean-field”.

Thank you, we added a dash as suggested.

6,
The y label of Figure 4a should be instead of D.
We have kept the plotted values and y-axis as D(sbar - s) rather than just (sbar - s) as this represents the spatial correction term to growth most directly.

Reviewer #2

I reviewed this paper together with a member of my lab, so this review represents the consensus of our perspectives.

The authors have made many valuable improvements to the paper, including a streamlined mathematical derivation of Eq. 6, testing the ML model on simulated data without spatial dependence as a sanity check, and further narrowing down the source of variation at the end of the results section. However, we recommend a few final revisions that address some remaining clarity issues:

1. We understand that the authors prefer to start their analysis with the ML model, but they motivate this in their introduction by stating that the ML model provides "insight" without saying specifically what that insight is. It seems like the main purpose of the ML model is to narrow down the features that need to be included in the mechanistic model (e.g., colony location, population size); if so, it would be helpful to state this explicitly. Or if we misunderstand, then it's even more important for the authors to better explain this.

Thank you for your suggestion. We are indeed mentioning the aim of the ML approach implicitly throughout the description, but to avoid confusion, we added now a sentence explicating it in the introduction of the ML approach. "*This allows us to determine the statistical importance of location when treated as an input feature and, at the same time, to obtain a general sense of how well population growth can be modelled using a set of chosen features.* ***That is, narrowing down the set of features (parameters) which we should incorporate into a mechanistic model, such as initial population size or location.***"

2. We appreciate the "rule of thumb" for comparing the spatial heterogeneity across nutrients that the authors have added to the result section. Since it seems that the difference in growth rate r_0 is driving the variation in the product $D r_0 \nu_1$, perhaps that can be an even simpler rule of thumb?

As a validation for their rule of thumb, the authors point to Fig. 4a. While we eventually saw that the plates with largest range on y-axis in Fig. 4a also have the highest product term $D r_0 \nu_1$ with the data from Table 1, this seemed unnecessarily subtle. Why not add a box plot of individual colony growth rates, lag times, and yields across the four plates? This figure would be similar to Fig. 2b, but showing the actual variation in the estimated traits themselves (rather than the explained variation). This figure would allow one to compare the nutrient conditions and the variation they introduce directly in a single panel.

Thank you, this is a helpful extra figure suggestion showing the variability of the raw data. Such a figure does indeed provide a neat way to contrast how the values to predict differ between environments. We added the suggested box plots as a new Figure 2b and moved the old Figure 2b to 2c. There we now have the raw data variability in these effective growth parameters and their ML interpretation side by side.

3. We see that the authors use the term "layer" to separate the colonies on each plate into five groups, depending on their distance to the nearest boundary. This makes sense, but needs to be explained more to the reader in the introducion when they show Fig. 1. Can the authors state the units of the "layer" variable in the caption to Fig. 1b? These units appear to be inconsistent across figures, as they range from 1-16 in Fig. 4a but 0.1-4 in Fig. 1b. It would also be helpful if the authors showed a schematic of a plate with the layer definition, for example by coloring the regions of the plate that belong to each layer (using a colony image similar to the first panel of Fig. 1d).

Thank you. The labels in those figures represented similar concepts but were defined somewhat differently. Clearly, this was confusing. We have changed the labels and colour scale of Figure 1b to be congruent with Figure 4a.

4. In Fig. 4a, the lines for layer 1 are too lightly colored to be easily visible, but this is the important layer for the reader to see here. Can the authors choose a color scheme with better contrast?

Thank you for the suggestion. The colour scheme has now changed for both Figure 1b and Figure 4a.

Reviewer #3

The authors have made significant improvements to the manuscript, addressing all of my comments. Below are two minor recommendations as a follow-up to the author's response to my original comments B and C.

Re: Comment B.

The authors have successfully strengthened the emphasis on their approach and incorporated the suggested references related to the coupling of population growth to the environment in the Discussion and Introduction sections. However, the abstract, as a concise summary of their findings, still maintains the original hierarchy of claims. To improve the abstract, I recommend that the authors also highlight the findings regarding temporal importance changes and their approach in the "Here we show..." sentence. If the authors state a good reason for keeping the current version (and the editor agrees with their rational), I can accept this decision.

Thank you. We have now changed the sentence to make a point about the temporal changes as well as the better understanding of the initial physiological state in causes for the variations.

Here we show that this variability can be attributed to the initial physiological states of the populations, the interplay between populations interacting with their local environment and the diffusion of nutrients and energy sources coupling the environments. We further show how the causes of this variability change throughout the growth of a population.

Re: Comment C.

I can follow the authors response to my initial comment. However, given the central role of the three different growth phases, I strongly recommend that the authors clearly indicate them in Fig. 2a (and ideally also in Fig. 1a. In addition, since the authors agreed with our notion that the so-called exponential phase is not clearly discernible as such, it would be ideal if they could add a short comment to explain why a true exponential behavior is not apparent in the data due to overlaying transients.

We have added a sentence where Figure 1a is discussed: *"The latter rates are of particular interest, as they differ substantially from the single constant value one would associate to exponential growth. For each plate there is a short such window, however, due to the variability of the start of growth of individual colonies, averaging blunts that region of constant growth which for individual colonies is also relatively short."*

We have added a schematic adjustment, exponential and deceleration phase to Figure 1a, as well as superposed a view of the relative growth rates $\rho_i(t)$ on the panels of Figure 2a to ease interpretation.
