# Supplementary material for: Quantifying massively parallel microbial growth with spatially mediated interactions
Source: PLoS Comput Biol. 2024 Jul 22;20(7):e1011585. doi: 10.1371/journal.pcbi.1011585 (PMC11293690; doi:10.1371/journal.pcbi.1011585)
Supplement: Attachment — Submitted filename: Comments to the authors_Borse et al.docx [file pcbi.1011585.s009.docx]

In this manuscript, the authors study the origin of spatially heterogeneous growth observed in massively parallel microbial colony assays. They follow a dual approach in which they first use a random forest machine learning approach to assess the relative importance of colony location and size for the prediction of relative growth rates under different conditions. Finding temporally varying contributions, they proceed to construct a series of mathematical models that gradually introduce more intricate couplings between population growth and the local environment to unravel the underlying dynamics.

In general, the paper is well-written and easy to follow. The approach and findings are well presented and of potentially significant interest to the wider scientific community. The scan-o-matic platform used to generate the experimental data in this manuscript belongs to a class of massively parallel growth assays that is widely used in the yeast genetics community and beyond. Positional artifacts are an inherent challenge of such assays and non-trivial to compensate for. While the isogenic implementation of the assay used in this work does not reflect the complexity of a typical assay, featuring a multitude of different genotypes and growth rates, it serves as a well-controlled test bed for the machine-learning-informed modeling approach.

Given the rapid rise of machine learning application in science, understanding how it can inform the design of interpretable mathematical models in complex (biological) systems is of major importance, not only to life sciences but to the scientific community in general. The well-controlled yeast-based model system investigated here is ideal to demonstrate the conceptual feasibility of the approach and showcase how key features of the observed growth dynamics can be rationalized in a meaningful way without detailed insights into the underlying mechanisms of colony expansion.

The authors claim to have shown how the coupling of population growth to their local environment and the interactions between environments can explain the observed spatial growth patterns. In addition, they claim to unravel how the temporal variations in relative importance of space and population size are coupled to different growth phases of the population. Both findings are supported by the presented data and analytical models. While the impact of resource diffusion on spatially structured growth may be of limited novelty, I find the presented machine learning approach conceptually relevant and the ensuing insights on temporally varying contributions of intra- and interpopulation insightful and important.

**Major Comments**

Comment A.

The experimental data presented in Fig. 1c shows very distinct identical patterns across all four plates (see e.g., areas around column 6-12, row 21-27 or column 10-22, row 2-9). Given that each of the four plates supposedly represent independent experiments, these patterns imply the presence of either experimental or data preprocessing artifacts. Please investigate and explain the origin of these patterns and correct your analysis pipeline accordingly. In addition, please provide access to the unprocessed raw data (currently missing).

This is of especially high importance since only one measurement per condition is given. Even though many colonies are imaged per time point, the entire ensemble of colonies only represents one data point per image (e.g. when calculating the importance of location) . If at all possible, including more technical replicates would greatly strengthen the statistical analysis of the presented claims. If this is not feasible, explain why and discuss the implications of these statistical limitations.

Comment B.

In the current structure of the manuscript, showing how spatial variability in growth can be attributed to couplings between population growth and local environments is presented as the main finding. Given the limited novelty of these insights (see e.g. refs. [1,2] below), I suggest that the authors reconsider the hierarchy of their claims, giving priority to their more interesting findings of temporal variations in location importance and the concept of their dual approach.

Comment C.

Throughout the manuscript, the authors several times allude to a biological context which they promise to discuss later. However, it was unclear to which part of the manuscript this referred to. It would be helpful if the authors could clearly indicate where this discussion can be found. Such a more thorough discussion of biological implications and potentially explanations for the phenomena observed in the data, such as different population growth phases, would greatly increase the relevance of the work for a more biological audience. For example, the exponential phase, mentioned several times, should imply a constant growth rate, yet no area of constant growth rate can be observed in Figure 1a.

Comment D.

Figure 4 is only briefly explained in one sentence in the main text and the figure caption. It would be beneficial if the authors could further elaborate on additional results from this figure and the conclusions drawn from them. Can you link the different behavior observed in the four environments to the underlying biology, such as different lag times, mode of colony expansion (exponential vs. boundary driven growth), etc.? A clear rationale for how different types of environments contribute to the overall findings (beyond demonstrating the robustness of the approach) would greatly help to motivate the design of the study.

**Minor Comments**

Comment E.

The introduction would benefit from a more thorough discussion of why interpretable models are so crucial, especially in the context of current challenges in explainable AI.

Comment F.

Please include a more detailed description of the experimental assay. An example of how the plate with colonies used in this experiment looks like (e.g., as part of Figure 1) would be ideal. Explain how *N*(*t*) is calculated. Is it a colony’s area or the estimated number of cells (taking into account the vertical dimension of yeast colonies)?

Comment G.

The order of plates seems arbitrary and confusing. Why this order? Ordering plate numbers with respect to growth speed and or conditions (e.g., gluc, gal, gluc+NaCl, gal+NaCl) would help the reader to keep track of conditions. Maybe even better: Substitute all plate references with the descriptive names while still using the aforementioned order in figures layouts

Comment H.

Units of actual time (min or h) for the x axis in Fig.1 would be more informative than time steps.

Comment I.

Explicitly explain how “importance” in Figure 2a is calculated. Given the prominence of this value for the message of the paper, merely pointing to the documentation of scikit-learn seems too vague.

Comment J.

Explain how Euclidian distance is calculated and why it is chosen as the error metrics in Figures 3 c and d.

Comment K.

Explain the parameter K in Figure 4.

Comment L.

On the right hand side of Equation (7) the derivative should be partial.

Comment M.

A main focus of the manuscript is the coupling of population size/growth to the local environment and the interaction between neighboring environments (see e.g. the third paragraph on page 12: “Thus, variability in growth across the populations of a plate is accounted for by the interactions between neighbouring populations resulting from this diffusion process.”). At the same time, the authors are clearly aware of the importance of boundary effects (in the same paragraph as above: “Due to the physical construction and layout of the plate, populations are exposed to a larger flow of nutrients and energy sources the further out in the grid they are located, which explains the variability pattern in growth across a plate.”). Yet, an explanation of how the two are linked (presumably via boundary effects on nutrient diffusion) is missing.

Naively, I would assume that boundary effects and initial nutrient consumption are the cause of the overall spatial gradient which is then dynamically modified by local consumer-resource dynamics, correct? Is the feedback on these local dynamics of positive or negative nature?

# References

# [1] Chacón, Jeremy M., Wolfram Möbius, and William R. Harcombe. "The spatial and metabolic basis of colony size variation." The ISME journal 12.3 (2018): 669-680.

[2] Baryshnikova, Anastasia, et al. "Quantitative analysis of fitness and genetic interactions in yeast on a genome scale." Nature methods 7.12 (2010): 1017-1024.
